# Separate and combined effects of Gua^+^ and SCN^−^ ions on charge carrier dynamics in mixed Sn–Pb perovskites

**DOI:** 10.1039/d5ta08016a

**Published:** 2026-01-26

**Authors:** Jasmeen Nespoli, Maartje J. van der Meer, Lara M. van der Poll, Xiaohui Liu, Tom J. Savenije

**Affiliations:** a Department of Chemical Engineering, Faculty of Applied Sciences, Delft University of Technology 2629 HZ Delft The Netherlands T.J.Savenije@tudelft.nl

## Abstract

Here, we investigated the separate and combined effects of guanidinium (Gua^+^) and/or thiocyanate (SCN^−^) ions on the opto-electronic properties of mixed Sn–Pb perovskites, which are used as absorber layers in photovoltaics. Therefore, we spin-coated Cs_0.25_FA_0.75_Sn_0.5_Pb_0.5_I_3_ thin films with GuaI, Pb(SCN)_2_ or GuaSCN, in the absence or presence of SnF_2_. By comparing the (micro)structural and opto-electronic properties of the perovskite films, we elucidated the functions of both ions. We found that SCN^−^ suppresses tin oxidation and doping, reduces crystal defects and improves the carrier transport properties, regardless of SnF_2_ addition. We demonstrate that this is due to coordination with Sn^2+^ and scavenging of Sn^4+^ in the spin-coating solution, resulting in a pile-up of SnO_*x*_ at the film surface. Gua^+^ is incorporated to a limited extent into the 3D cubic perovskite structure. Gua^+^ cannot suppress tin oxidation and doping, making this additive useless without SnF_2_. Conversely, when combined with SnF_2_, Gua^+^ enhances the carrier transport properties. Combining Gua^+^ and SCN^−^ until a maximum addition of 4 mol% and SnF_2_ results in large grains and pinhole-free films with superior charge carrier transport properties, leading to a substantial increase in the pseudo-open circuit voltage of 50 mV. Addition of >4 mol% GuaSCN leads to the formation of Gua-based 2D perovskites, including Gua_*x*_FA_2−*x*_Sn_*y*_Pb_1−*y*_I_4_ and Gua_*x*_FA_3−*x*_Sn_*y*_Pb_2−*y*_I_7_, which do not improve the carrier dynamics. In short, we observe a synergistic effect on addition of Gua^+^ and SCN^−^ ions, which leads to improved structural and opto-electronic properties, which is promising for implementation in solar cells.

## Abstract 2

Guanidinium and thiocyanate ions show a synergistic effect in mixed tin-lead perovskites, passivating crystal defects, enhancing carrier transport, and yielding pinhole-free films with large grains.

## Graphical abstract

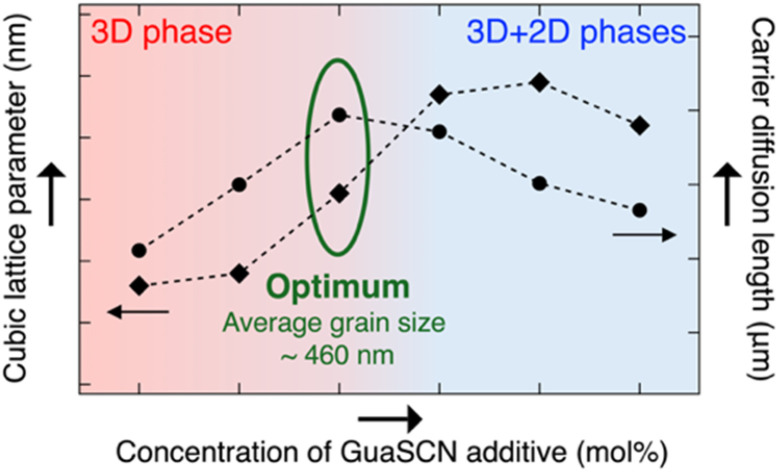

## Introduction

Metal halide perovskites (MHPs) have been used in photovoltaics since 2009 and in less than 16 years they became the fastest advancing photovoltaic technology.^1,2^ MHPs have an ABX_3_ structure, where the A-sites are commonly occupied by cations such as methylammonium (MA^+^: CH_3_NH_3_^+^), formamidinium (FA^+^: HC(NH_2_)_2_^+^), or cesium, Cs^+^, while iodide, I^−^, and bromide, Br^−^, are commonly chosen for the X-sites.^3^ Sn–Pb perovskites present a mixture of Sn^2+^ and Pb^2+^ at the B-sites, which results in a low bandgap of 1.2–1.6 eV depending on the ratio.^3^ Sn–Pb perovskite thin films can be used as absorber layers in single-junction solar cells to achieve power conversion efficiencies close to 24%,^4,5^ or in bottom cells in all-perovskite multi-junction devices to go beyond 28%.^6,7^

Counteracting the oxidation of Sn^2+^ to Sn^4+^ was shown to improve the performance of Sn–Pb perovskite solar cells. Tin oxidation mostly induced by oxygen^8–12^ causes p-doping and crystal defects, which increase the charge carrier recombination and reduce the mobility–lifetime product.^11–15^ SnF_2_ is a widely used additive to counteract these effects *via* a ligand exchange reaction with SnI_4_ in the precursor solution.^15–20^ However, this additive also introduces compositional heterogeneities at the surface of the films, where there is a pile-up of Sn^4+^ products,^17–19^ as also shown in our previous work.^20^ Other additives capable of modifying the structure and microstructure of Sn–Pb perovskites have been explored. Among these, guanidinium thiocyanate (GuaSCN) has emerged as a promising supplementary additive to suppress tin oxidation, doping and crystal defects more effectively than only SnF_2_.^21–23^ It is claimed that GuaSCN can passivate traps, elongate carrier lifetimes, improve the film morphology and protect the films from oxygen.^21–24^ Furthermore, it is reported that the individual guanidinium (Gua^+^) or thiocyanate (SCN^−^) ion can also increase the charge carrier lifetimes in Sn-containing perovskites even more than SnF_2_ alone.^25–28^ This indicates that Gua^+^ and/or SCN^−^ may passivate some other defects, which are not targeted by SnF_2_, and that the mechanism is probably different. We suppose that Gua^+^ and SCN^−^ ions influence the perovskite properties through distinct but possibly synergistic mechanisms. Based on the literature, the functionality of Gua^+^ is expected to emerge in the solid crystal structure. Gua^+^ may be incorporated into the A sites and affect the crystallization and morphology of the film,^21,22,29,30^ as well as potentially suppress tin oxidation, enable doping and passivate defects.^21,22,26,31^ Hence, the actual role of Gua^+^ is not well specified in the literature. At the same time, as described by Goldschmidt's rule, which predicts the dimensionality of ABX_3_ perovskite structures on the basis of geometrical factors, considering the ionic radii of the A, B and X constituents, low-dimensional perovskite phases may form when a large-sized cation as Gua^+^ is present.^29^ In fact, it has been reported that addition of GuaSCN leads to low-dimensional (1D, 2D) Gua-containing perovskite phases (alongside 3D perovskite), which are claimed to be beneficial for carrier transport, due to defect passivation and faster charge extraction.^21,22^ On the other hand, SCN^−^ is expected to play a role both in the precursor solution and during the film crystallization and decrease doping and defects.^21,22,28,32^ Similarly to Gua^+^, the actual function of SCN^−^ is not yet fully clarified in the literature.

However, in the literature on Sn-containing perovskites, there is a lack of clarity and consistency regarding the individual roles of Gua^+^ and SCN^−^ ions, as well as their combined effect. Often the effect of only one type of additive, such as GuaI,^25^ GuaBr,^26^ KSCN,^33^ MASCN,^34^ FASCN,^27^ Sn(SCN)_2_,^28^ or Pb(SCN)_2_,^35^ has been studied, preventing a comparison of the separate effects of Gua^+^ and SCN^−^ on the same perovskite. On the other hand, when these additives have been studied and compared in full solar cell devices, the specific effects at the level of the structural and opto-electronic properties of the perovskite have not been investigated.^21,23^ Although the effects of Gua^+^- and/or SCN^−^-containing additives have been studied in some reports, they are almost always found in combination with other additives such as SnF_2_,^21–23,25–28^ metallic Sn^0^ powder,^23,25^ or large cations, *e.g.* phenylethylammonium (PEA^+^: C_6_H_5_CH(NH_2_)CH_3_^+^),^22,23,25,27,34^ which complicates the analysis. The lack of basic knowledge on this topic calls for a study of the precise functions of Gua- and/or SCN-containing additives. To shed light on this, we studied the separate and combined effects of Gua^+^ and/or SCN^−^ ions on spin-coated Cs_0.25_FA_0.75_Sn_0.5_Pb_0.5_I_3_ perovskite thin films. Our work investigates whether changes can be assigned to the single ions alone, *i.e.* Gua^+^ or SCN^−^, or additional effects emerge when both ions are present simultaneously, *i.e.* in the form of the combined additive GuaSCN. We also examined the collective effect of each additive in the absence or in the presence of SnF_2_, used by nearly all research groups around the world, to disentangle the role of Gua^+^ and/or SCN^−^ ions from that of SnF_2_. For this, we deposited Cs_0.25_FA_0.75_Sn_0.5_Pb_0.5_I_3_ perovskite thin films with different additives on quartz substrates using the antisolvent spin-coating method. We chose GuaI and Pb(SCN)_2_ as additives to avoid introducing foreign ions and thereby increasing the complexity of the system. While the choice of GuaI is obvious, Pb(SCN)_2_ was used as the SCN^−^ source because (i) Sn(SCN)_2_ could have introduced additional complications related to tin oxidation and (ii) FASCN could have altered the A-sites, which are instead targeted specifically by the Gua-containing additive. We acknowledge that even small deviations (0.5–1%) from the ideal perovskite precursor stoichiometry can alter the relative device performance.^36^ However, to avoid making the systems even more complex by choosing foreign cations, we selected counterions already present in the precursor solution.

**Scheme 1:**
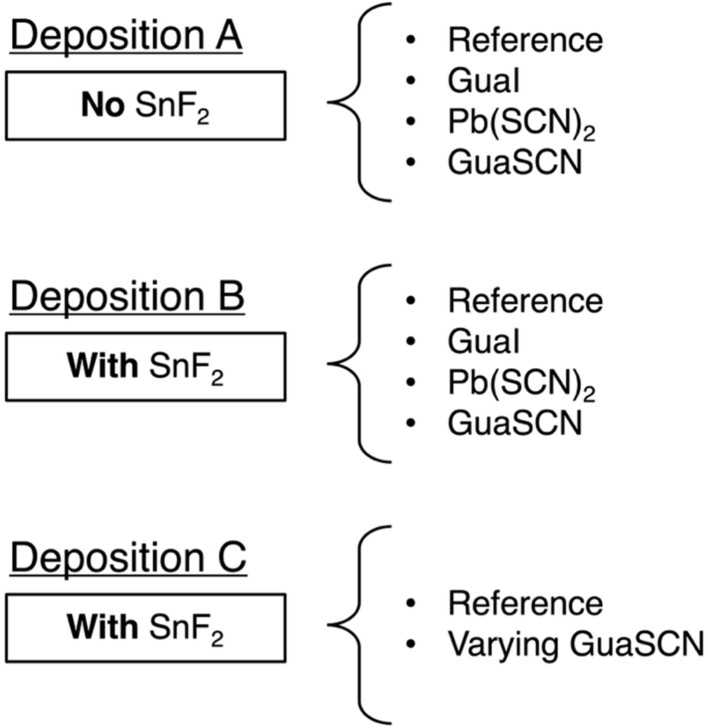
Schematic diagram of the three deposition sets A, B and C of mixed Sn–Pb perovskites, where each deposition uses either nothing, GuaI, Pb(SCN)_2_ or GuaSCN as an additive.

We started with a parent solution of Cs_0.25_FA_0.75_Sn_0.5_Pb_0.5_I_3_ perovskite to which we added small volumes of either GuaI, Pb(SCN)_2_ or GuaSCN, in the absence (deposition A) or in the presence (deposition B) of SnF_2_, or varying small volumes of GuaSCN in the presence of SnF_2_ (deposition C). These procedures yielded the following spin-coating solutions for three sets of depositions given in Scheme 1.

For each deposition, we investigated the structural and microstructural properties of the perovskite layers by X-ray diffraction (XRD) and scanning electron microscopy (SEM), respectively. Additionally, we studied the compositional properties at the surface and in the bulk of the layer by X-ray photoelectron spectroscopy (XPS). We also examined the optical properties by UV-Vis-NIR spectroscopy (UV-Vis). We analyzed the conductivity of the films in the dark and under illumination by microwave-based techniques, namely steady-state microwave conductance (SSMC) and time-resolved microwave conductivity (TRMC). By comparing these perovskite films, we elucidated the function of each additive, crucial for optimizing the perovskite properties and obtaining absorber layers for, *e.g.*, highly efficient Sn–Pb perovskite solar cells.

## Experimental section

### Materials

Cesium iodide (CsI, 99.999%) and tin(ii) fluoride (SnF_2_, 99%) were purchased from Merck-Sigma Aldrich. The organic halide salt formamidinium (FAI, 99.99%) was purchased from Greatcell Solar Materials. Lead(ii) iodide (PbI_2_, 99%) was purchased from Acros Organics. Tin(ii) iodide (SnI_2_, 99.999%, 10 mesh beads) and tin(iv) iodide (SnI_4_, 99.998%, ultradry) were purchased from Thermo Scientific Chemicals – Alfa Aesar. The powder of SnI_2_ was obtained by grinding the SnI_2_ beads using a pestle and a mortar. Guanidinium thiocyanate (GuaSCN, ≥99.0%), guanidinium iodide (GuaI, ≥99%), lead thiocyanate (Pb(SCN)_2_, 99.5%), dimethylformamide (DMF, anhydrous, 99.8%), dimethyl sulfoxide (DMSO, anhydrous, ≥99.9%) and anisole (anhydrous, 99.7%) were purchased from Merck-Sigma Aldrich.

### Synthesis

Quartz substrates were cleaned using an ultrasonic bath (5 min in acetone + 5 min in isopropanol) and UV–ozone treatment for 10 min. In a glovebox with low levels of O_2_ ≲ 0.5 ppm and H_2_O ≃ 0.8 ppm, two solutions (1.6 M) of pure Pb-based and pure Sn-based perovskites (Cs_0.25_FA_0.75_PbI_3_ and Cs_0.25_FA_0.75_SnI_3_) were prepared by stirring overnight the perovskite precursors in DMF and DMSO with a volumetric ratio of 4 : 1. Moreover, solutions of GuaSCN (0.7 M), GuaI (0.7 M), Pb(SCN)_2_ (0.7 M) and SnF_2_ (0.5 M) were prepared by stirring GuaSCN, GuaI, and Pb(SCN)_2_ powders overnight in DMF/DMSO (as for the perovskite solutions) and SnF_2_ powder in only DMSO. The Pb(SCN)_2_ and SnF_2_ solutions were stirred again for 15 min at 50 °C on the following day, to ensure full dissolution before spin-coating. The parent solution of Cs_0.25_FA_0.75_Sn_0.5_Pb_0.5_I_3_ perovskite was obtained by mixing equal volumes of the two Cs_0.25_FA_0.75_PbI_3_ and Cs_0.25_FA_0.75_SnI_3_ perovskite solutions. Small volumes of solutions of the different additives were added to the parent Cs_0.25_FA_0.75_Sn_0.5_Pb_0.5_I_3_ perovskite solution and mixed for ∼1 h 30 min. To study the separate and combined effects of Gua^+^ and SCN^−^ ions and disentangle the role of the widely used SnF_2_ from these other additives, spin-coating solutions with varying additives were prepared for three depositions as described in Scheme 1. The mixed Sn–Pb perovskite thin films with different additives were deposited by antisolvent spin-coating on the quartz substrates. The perovskite solutions were dripped evenly onto the substrate and spin coated with an initial rotational acceleration ramp of 500 rpm s^−1^ and a final speed of 3000 rpm for 60 s. After 50 s from the beginning of the rotation, 200 µL of anisole (antisolvent) were poured gently but firmly in ≤1 s from approximately 1–1.5 cm above the surface of the sample. Lastly, annealing at 100 °C for 10 min was performed immediately afterwards.

### X-ray diffraction (XRD)

The XRD analysis of the films was carried out by using a Bruker D8 Advance-ECO X-ray diffractometer, equipped with a Cu-K_α_ X-ray source (*λ* = 1.542 Å) operating at 40 kV and 25 mA and a Lynxeye-XE-T 1D position-sensitive energy-discriminative detector. The measurements were carried out in Bragg–Brentano geometry with a fixed sample illumination of 5.0 mm for a range of angles 2*θ* = 5–60°, a step size of 0.01° and a measuring time of 0.01 s per step. The reference XRD patterns were simulated by using DIFFRAC.EVA V6.0 Bruker software from .cif files retrieved from the literature and databases.^37–46^ This software was also used to determine the lattice parameter of the 3D cubic perovskite phase, *a*, by tuning the unit cell parameter in a reference XRD pattern. For the analysis of the Gua-containing 2D phases, the interplanar distance, *d*_*hkl*_, was calculated from the XRD peaks positions (*i.e.* the diffraction angles, 2*θ*) in the diffraction patterns by using Bragg's law and the wavelength of the Cu-K_α_ X-rays, namely *λ* = 1.54056 Å. Moreover, the Gua-containing 2D crystal structures were simulated from the .cif files mentioned above by using VESTA software.

### Scanning electron microscopy (SEM) with energy dispersive X-rays (EDX)

A JEOL JSM-IT700HR field effect scanning electron microscope was used to obtain top view images of the films and analyze their elemental composition. SEM images were obtained by probing secondary electrons (SEs) and back-scattered electrons (BSEs) with, respectively, an Everhart–Thornley (ET) type SE detector and a BSE detector for high vacuum observation in the chamber, with the SEM operated at 3 kV and 30 pA. The average grain size and distribution were calculated from SE images obtained over 400 distances by using ImageJ software and then fitting with a Gaussian function. Spatially resolved SEM/EDX was conducted by using a 30 mm^2^ SDD detector (liquid nitrogen free). EDX elemental maps were collected at 15 kV and 50 pA, scanning an area of ∼123 µm^2^. The effective spatial resolution of back-scattered electrons and X-rays is limited to a few micrometers by the interaction volume in the perovskite films.

### X-ray photoelectron spectroscopy (XPS)

The elemental composition and chemical state analyses of the films were carried out by using a Thermo Scientific K-Alpha system for XPS, incorporating an X-ray gun based on an Al K_α_ radiation source with an energy of 1486 eV and a spot size kept at the default value of 800 × 400 µm^2^. The samples were transferred to the XPS setup by means of an air-tight sample holder and a load lock. All measurements were conducted under high vacuum conditions (*P* < 4 × 10^−7^ mbar). A flood gun operating at 0.15 mA and 1 V was used to replenish the electrons emitted from the sample surface from the system to hinder charging during the measurement. The surface XPS scans are performed prior to any etching to avoid damage by the Ar^+^ sputter gun. The XPS peaks were rescaled to the reference peak at *E*_b_ ∼ 284.8 eV in the XPS surface analysis for the C 1s core levels, corresponding to the adventitious C–C chemical state. The bulk XPS scans were obtained after etching for 30 s the thin film with an argon-based ion beam with energy *E* = 1 keV.

### UV-Vis-NIR spectroscopy (UV-Vis)

The optical absorption properties (absorptance) of the films were measured using a PerkinElmer LAMBDA 1050+ UV/Vis/NIR spectrophotometer with a 150 mm integrating sphere. The absorption (optical density, O.D.) of solutions was measured using a PerkinElmer LAMBDA 365 UV/Vis spectrophotometer by using quartz cuvettes with an optical path length of 0.20 cm.

### Steady-state microwave conductance (SSMC)

SSMC measurements to study the dark conductivity, *i.e.* the doping level, of the perovskite thin films were performed in the dark and under N_2_. The microwaves (frequencies between 8.2 and 12.2 GHz) pass through the film located in the microwave cavity cell partially closed with an iris. At the resonant frequency (∼8.5 GHz), a standing wave forms in the cavity and the maximum of the microwave electric field overlaps with the film. The microwaves are partially absorbed due to the interaction with free, mobile charge carriers and partially reflected. This causes a loss of microwave power (Δ*P*), resulting in a dip at the resonant frequency in the microwave frequency scan. The dip is expressed in *R*_0_ and denotes the fraction of reflected microwave power in comparison to a fully reflecting end plate. The normalized microwave power loss signal (Δ*P*/*P*), *i.e.* the resonant frequency dip, can be simulated to calculate *σ*_dark_. The SSMC measurements are reliable and reproducible because of the fixed sample positioning, microwave cavity dimensions, and iris size, which keep the coupling and quality factor constant. The error estimation is ±∼1% for multiple measurements performed on the same sample and ±∼5% for measurements performed on several samples of the same deposition.

### Time-resolved microwave conductivity (TRMC)

TRMC measurements were performed to study the charge carrier dynamics and transport properties in the perovskite thin films. A pulsed Nd:YAG laser is used to excite charge carriers in the films by pulses of the duration of ∼3.5 ns at a repetition of 10 Hz and wavelength *λ* = 800 nm. The laser intensity is tuned between 10^10^ and 10^13^ photons cm^−2^ by using an array of neutral density filters. During a TRMC measurement, the microwaves pass through the perovskite film mounted in a microwave cavity cell partially closed with an iris (which features an instrumental response time of 18 ns), where they are partially absorbed due to the interaction with free, mobile photogenerated carriers. A circulator separates the incident from the reflected microwaves and the loss in microwave power between the reflected and the incident microwave is recorded as a function of the time elapsed after the laser pulse (Δ*P*(*t*)). This is related by the sensitivity factor *K* (calculated as reported in a previous study)^20^ for the microwave cavity cell to the time-resolved change in photoconductance between dark and after illumination (Δ*G*(*t*)), *i.e.* the transient photoconductance signal. For TRMC, the error estimation is ±∼5% for both multiple measurements performed on the same sample and measurements performed on several samples of the same deposition. The maximum TRMC signal, normalized by the intensity of the laser, *I*_0_, and the absorbed fraction of light at the excitation wavelength, *F*_A,_ and a microwave cell form factor, *β*, can be expressed by the product of charge carrier yield, *φ*, and the sum of gigahertz-frequency mobilities. We assumed *φ* = 1 for direct bandgap perovskites with low exciton binding energy at room temperature. It follows that Δ*G*_max_/*βeI*_0_*F*_A_ = Σ*µ*. The carrier lifetime, *τ*_1/2_, is calculated as the time to reach half of the maximum TRMC signal, recorded at the same laser intensity for all perovskite films. The carrier diffusion length, *L*_D_, was calculated as *L*_D_ = √(*Dτ*_1/2_), where *D* is the carrier diffusion coefficient. This is calculated as *D* = *k*_B_*Tµ*/*e*, where *k*_B_ is the Boltzmann constant, *T* is the temperature, *µ* is the carrier mobility and *e* is the elementary charge.

### Microwave-based quasi-Fermi level splitting measurements and derivation of pseudo-*J*–*V* curves

The microwave conductivity setup was also used to determine the quasi-Fermi level splitting (QFLS) of the perovskite films. The QFLS was determined by illuminating the perovskite layer using a monochromatic green LED (*λ* = 522 nm) to create photo-induced excess charge carriers. By integrating the solar spectrum from higher photon energies down to the bandgap energy of ∼1.26 eV of our Cs_0.25_FA_0.75_Sn_0.5_Pb_0.5_I_3_ perovskite films,^20^ a photon flux of ∼2.3 × 10^17^ photons per s per cm^2^ was found, representing 1 sun intensity. The QFLS can be calculated using eqn (1).^47^1
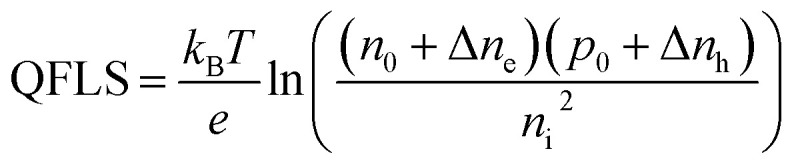
where *k*_B_*T*/*e* is the thermal voltage, *n*_i_ represents the intrinsic carrier density, *n*_0_ and *p*_0_ are respectively the dark electron and dark hole densities in thermal equilibrium, and Δ*n*_e_ and Δ*n*_h_ are respectively the photo-induced excess charge carrier densities. For our Cs_0.25_FA_0.75_Sn_0.5_Pb_0.5_I_3_ perovskite films, we calculated *n*_i_ ∼ 1 × 10^9^ cm^−3^ from the bandgap energy of ∼1.26 eV.^20^ To simplify eqn (1), we assumed that *n*_0_ is close to 0 for a p-type semiconductor like Sn-containing perovskites. Additionally, since the absorption of each photon leads to the generation of one free electron and one free hole, we considered Δ*n*_e_ = Δ*n*_h_ = Δ*n*. The pseudo-*JV* curves were constructed from QFLS measurements determined at various light intensities and following reported procedures.^48,49^ The QFLS was directly used as the voltage, while the current was derived from the LED light intensities employed in the measurement. The pseudo-short circuit current density, *pJ*_SC_, was estimated from the Shockley–Queisser limit for our Cs_0.25_FA_0.75_Sn_0.5_Pb_0.5_I_3_ perovskite films with a bandgap of ∼1.26 eV,^20,50^ and considering a generation efficiency of 90% of the radiative limit.^49^ The resulting pseudo-*J*–*V* curves were obtained with the determined *pJ*_SC_ ∼ 33.8 mA cm^−2^.

We underline that to minimize the detrimental effect of exposure to ambient air, the perovskite thin films were transferred to the characterization setups by means of an N_2_-filled air-tight transfer unit and immediately measured after being removed from it. More specifically, all SSMC and TRMC measurements were done by sealing the microwave cells under N_2_ in a glovebox. For the XPS measurements, a vacuum transfer module specifically designed for the XPS system was used to transfer the layers in the setup.

## Results and discussion

**Fig. 1:**
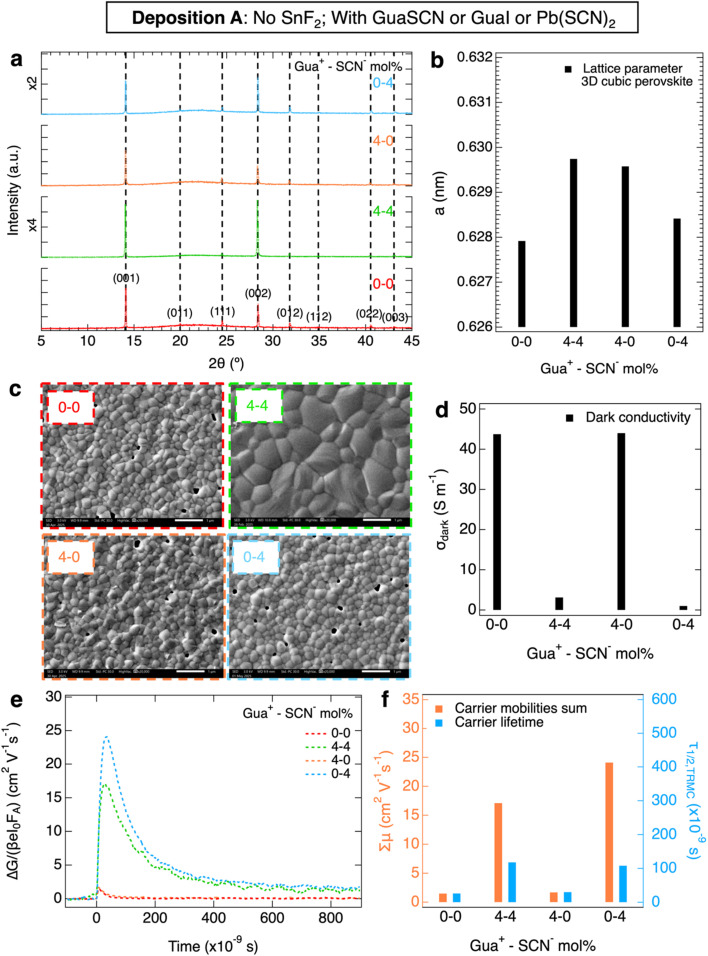
Separate and combined contributions of Gua^+^ and SCN^−^ ions to the structural and charge carrier properties, upon addition of 0 and 4 mol% Gua^+^ and SCN^−^ ions and 0 mol% SnF_2_. In (a), the XRD shows peaks corresponding to a 3D cubic perovskite phase (black dashed lines and Miller indices). To facilitate the comparison, note that the XRD intensity axes were rescaled by factors of ×2 and ×4 (zoomed out) for the layers with high-intensity peaks containing 4 mol% SCN^−^ and 4 mol% GuaSCN, respectively. In (b), the lattice parameter, *a*, of the 3D cubic perovskite phase is shown. In (c), the SEM images were taken at 20 000× magnification. Regarding the charge carrier properties, (d) dark conductivity, *σ*_dark_, (e) TRMC photoconductivity transients obtained with laser excitations *λ* = 800 nm at the same intensity and (f) sum of carrier mobilities, Σ*µ*, and lifetimes, *τ*_1/2_, are shown.

First, we studied the perovskite layers from deposition A, by comparing the reference film without any additives to films with 4 mol% Gua^+^ and/or SCN^−^ w.r.t. (CsI + FAI) addition, but without SnF_2_. We investigated the structural properties of these layers by XRD shown in Fig. 1a. More details about the perovskite synthesis are provided in the SI. We note that independently of the additive, the 3D cubic perovskite phase forms without any signs of other crystalline phases. The addition of only Gua^+^ or GuaSCN always causes a small but reproducible shift of 0.05–0.10° to lower diffraction angles (in detail in Fig. S1a–c), implying a slightly larger lattice parameter, *a*, as shown in Fig. 1b. We attribute this expansion of the unit cell to a minor incorporation of Gua^+^ in the crystal. Gua^+^ has a molecular radius of 278 pm and can occupy the A sites of the ABX_3_ cubic perovskite structure by replacing the smaller FA^+^ (253 pm) or Cs^+^ (167 pm).^29^ However, we expect that only a small fraction of the A sites is substituted by Gua^+^ since otherwise a collapse from a 3D to a lower-dimensional perovskite structure occurs,^29^ as we will also show in deposition C. Incorporation of Gua^+^ in the cubic perovskite crystal is also mentioned in the literature,^30^ albeit often at higher Gua^+^ concentrations.^21,25,26^ In short, from our XRD measurements, the incorporation of only Gua^+^ does not substantially improve the crystallinity but results in a small lattice unit expansion.

In contrast to the addition of only Gua^+^, the addition of SCN^−^ or GuaSCN results in high-intensity XRD peaks in comparison to the other layers, as visible in Fig. 1a. The large intensity peaks suggest high crystallinity, in agreement with the small full-width half maximum (FWHM) of the XRD peaks (Fig. S1d), and a strong preferential crystal orientation along the [001] direction for both films with SCN^−^ or GuaSCN. In detail, the (001)/(111) peak intensity ratio of the film with GuaSCN is ∼3 times higher than that of the other films. The SEM images in Fig. 1c show the polycrystalline nature of the spin-coated Sn–Pb perovskite layers. There is a negligible effect on the microstructure when only Gua^+^ or only SCN^−^ is added. In contrast, by combining Gua^+^ and SCN^−^ the average grain size, <*D*>, triplicates in comparison to the other films, resulting in a layer with <*D*> ∼ 750 nm and no pinholes. Hence, we conclude that addition of SCN^−^ or of GuaSCN improves the crystallinity substantially, but only in the case of GuaSCN significantly are larger grains and fewer grain boundaries (GBs) observed.

We also performed XPS on the perovskite films from deposition A to reveal the elemental composition. Interestingly, we noticed from surface and bulk XPS scans on the O 1s orbitals, shown in Fig. S24, that the addition of only SCN^−^ results in a strong accumulation of SnO_*x*_ at the film surface. This suggests that SCN^−^ scavenges Sn^4+^ and results in SnO_*x*_ at the film surface during crystallization, which in turn limits the formation of bulk tin vacancies and doping.^18–20^

Next, we investigated the opto-electronic properties of the perovskite layers by UV-Vis. These measurements show that the studied additives do not affect the absorption onset (Fig. S5a), in line with the literature for these additives at low concentrations.^21,22,25,27,30^

The films of deposition A were also studied by microwave-based characterization techniques, which are based on the absorption of microwaves by free, mobile charge carriers in the perovskites. We performed SSMC on the films mounted in a microwave cavity cell under N_2_ to determine the dark conductivity, *σ*_dark_. At the resonant frequency a standing wave is formed in the cavity, whose maximum electric field overlaps with the sample and maximizes the interaction. This frequency (∼8.5 GHz) can be found by recording a frequency scan and observing the resulting dip in the microwave reflection frequency scan.^51^ When the perovskite layer has high *σ*_dark_, due to doping, the microwave absorption is enhanced, and the dip gets deeper. Then, we quantified *σ*_dark_ from the frequency dips in Fig. S6a by following the method described in our previous work.^20^ In Fig. 1d, we observe that the reference film has a high *σ*_dark_ > 40 S m^−1^, attributed to doping resulting from tin oxidation.^15,20,52^ The addition of only Gua^+^ does not affect *σ*_dark_, which is comparable to that of the reference film. This is in contrast with other studies reporting lower tin oxidation, doping and defect density in Sn–Pb perovskites, which was related to the formation of low-dimensional Gua-based perovskites.^21,22,26^ Conversely, the presence of SCN^−^ or GuaSCN results in lower *σ*_dark_, resulting in nearly intrinsic films with *σ*_dark_ ≤ 3.1 S m^−1^. Obviously the SCN^−^ ion is able to effectively scavenge Sn^4+^ from the spin coating solution, which we will discuss in detail later on.

Furthermore, we performed TRMC to study the photoinduced charge carrier dynamics in the perovskites. For TRMC measurements, a nanosecond pulsed laser is used to generate excess charge carriers in the film located in the microwave cell described above. The photoconductance transient shows a fast increase upon photoexcitation, followed by a decay due to charge carrier immobilization in traps and recombination. Moreover, the maximum TRMC signal is related to the product of the sum of carrier mobilities, Σ*µ*, and the charge carrier yield, *φ*. More details about the TRMC technique can be found in previous reports.^51,53^ Samples of deposition A were excited with laser pulses with a wavelength of *λ* = 800 nm and the resulting photoconductance traces (see Fig. S7) are shown in Fig. 1e. From these traces the carrier mobilities were determined assuming *φ* = 1 and half lifetimes were extracted and are plotted in Fig. 1f. The reference film and the film with only Gua^+^ present poor photoinduced carrier transport properties, *i.e.* a low mobility–lifetime product, resulting in a short carrier diffusion length *L*_D_ ∼ 250 nm. Most importantly, the films with either SCN^−^ or GuaSCN show enhanced mobility–lifetime products and much longer *L*_D_ values of ∼2 µm (Fig. S10a). This can be explained by the high *σ*_dark_ of the samples without either SCN^−^ or GuaSCN. As discussed above, tin oxidation leads to doping, crystal defects and deep traps in the Sn-containing perovskites.^12,54^ Due to ionized impurity scattering, the mobilities of photoexcited carriers will be lower. In addition, the high concentration of dark carriers leads to rapid recombination with the excited carriers, reducing the charge carrier lifetimes. Both aspects will lead to reduced *L*_D._ Finally, we observe no clear correlation between the grain size and the carrier transport properties. This can be attributed to the fact that TRMC maps the local conductivity in the polycrystalline perovskite film and is hardly affected by long-distance carrier transport. Hence, the films prepared with only SCN^−^ or with GuaSCN demonstrate long comparable *L*_D_ values, while only for the latter the grain size of the films has increased substantially.

**Fig. 2:**
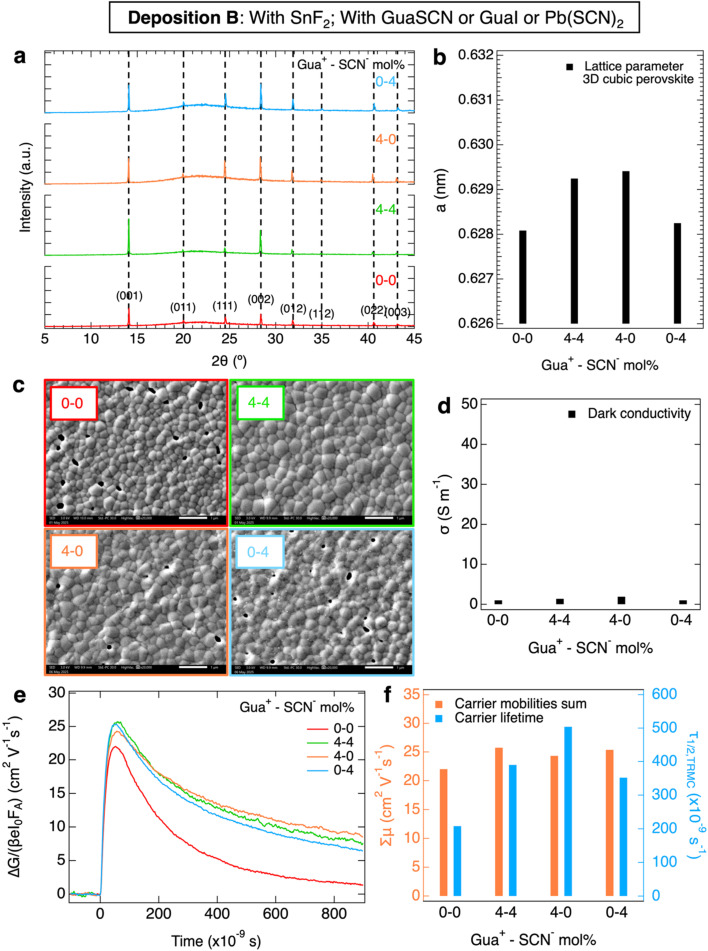
Separate and combined contributions of Gua^+^ and SCN^−^ ions to the microstructural and charge carrier properties upon addition of 0 and 4 mol% Gua^+^ and SCN^−^ ions and 10 mol% SnF_2_. In (a), the XRD pattern shows peaks corresponding to a 3D cubic perovskite phase (black dashed lines and Miller indices). In (b), the lattice parameter, *a*, of the 3D cubic perovskite phase is also shown. In (c), the SEM images were taken at 20 000× magnification. Regarding the charge carrier properties, the (d) dark conductivity, *σ*_dark_, (e) TRMC photoconductivity transients obtained with laser excitations *λ* = 800 nm at the same intensity and (f) sum of carrier mobilities, Σ*µ*, and lifetimes, *τ*_1/2_, are shown.

Then, we studied in Fig. 2 the perovskite layers from deposition B, now all with 10 mol% SnF_2_ w.r.t. SnI_2_ addition. Again, we compared the reference film to films with 4 mol% Gua^+^ and/or SCN^−^, with the aim of investigating the effect of the commonly used SnF_2_ in combination with Gua^+^ and/or SCN^−^. From the structural analysis of the XRD patterns shown in Fig. 2a, we conclude that all layers are in the same cubic 3D perovskite phase. No clear improvement in crystallinity is visible from the FWHM of the XRD peaks (Fig. S2d). Analogously to Fig. 1a, we observe a shift to lower angles of the XRD peaks for layers with Gua^+^ or GuaSCN (in detail in Fig. S2a–c) Again, we attribute this shift to the expansion of lattice parameter *a*, shown in Fig. 2b, due to the incorporation of a small fraction of Gua^+^. As shown in the SEM images in Fig. 2c, S13 and S14, the grain size increases slightly, from <*D*> ∼ 250 nm of the reference film to ∼300 nm if only Gua^+^ or SCN^−^ is added. The largest <*D*> is obtained for perovskite layers with GuaSCN, leading to a layer with grains of <*D*> ∼ 330 nm and no visible pinholes, similar to our previous observations for perovskite layers with GuaSCN but no SnF_2_. However, we note that for these layers with SnF_2_ the grains do not grow as much as in the film without SnF_2_, which suggests that the grain growth is limited with SnF_2_. This might be related to the higher concentration of Sn^2+^ which speeds up the nucleation rate during the film crystallization from solution.^55–57^ From the XPS elemental analysis of the O 1s surface and bulk scans, shown in Fig. S25, we noticed that not only SCN^−^, but also SnF_2_ scavenges Sn^4+^, leading to SnO_*x*_ at the film surface, as observed in our previous work and reported elsewhere.^17–20^ Indeed, the film with a combination of SCN^−^ and SnF_2_ shows the strongest accumulation of SnO_*x*_ at the surface. Interestingly, the SEM images of these layers at 50 000× magnification in Fig. S17 show minuscule particles on the surface of the layer with 4 mol% SCN^−^ and 10 mol% SnF_2_, accumulating at the GBs, which we attribute to SnO_*x*_.

Moreover, we studied the opto-electronic properties of these perovskite layers, which show absorption onsets similar to the samples with no SnF_2_ addition (Fig. S5b). We performed SSMC measurements (Fig. S6b), which revealed that all layers with 10 mol% SnF_2_ exhibit a nearly intrinsic behavior with low *σ*_dark_ ≤ 1.9 S m^−1^ as shown in Fig. 2d, regardless of the other additives. This is expected, as SnF_2_ is known to scavenge Sn^4+^ and prevent doping.^15–18,20^ In Fig. 2e, we compare the TRMC traces recorded on pulsed excitation at the same intensity and wavelength, from which we derive the corresponding carrier transport properties, shown in Fig. 2f. In comparison to the reference layer, which exhibits Σ*µ* ∼ 22 cm^2^ V^−1^ s^−1^ and *τ*_1/2_ ∼ 210 ns, we observe that all samples with Gua^+^ and/or SCN^−^ show slightly higher mobilities of Σ*µ* ∼ 25 cm^2^ V^−1^ s^−1^. Interestingly, independent of the additive, the carrier lifetimes improve more significantly to *τ*_1/2_ ≥ 350 ns. As a result, compared to the reference layer with an *L*_D_ of ∼2.5 µm, all samples with Gua^+^ and/or SCN^−^ ions have an improved but similar *L*_D_ of ∼3.5 µm (Fig. S10b).

From deposition B, we conclude that with SnF_2_ the separate or combined effects of Gua^+^ and/or SCN^−^ result in similar enhancements of the carrier transport properties. Despite small structural variations, the only remarkable difference is in the microstructure. In fact, only the addition of GuaSCN and SnF_2_ leads to a perovskite film with larger grains and no pinholes.

**Fig. 3:**
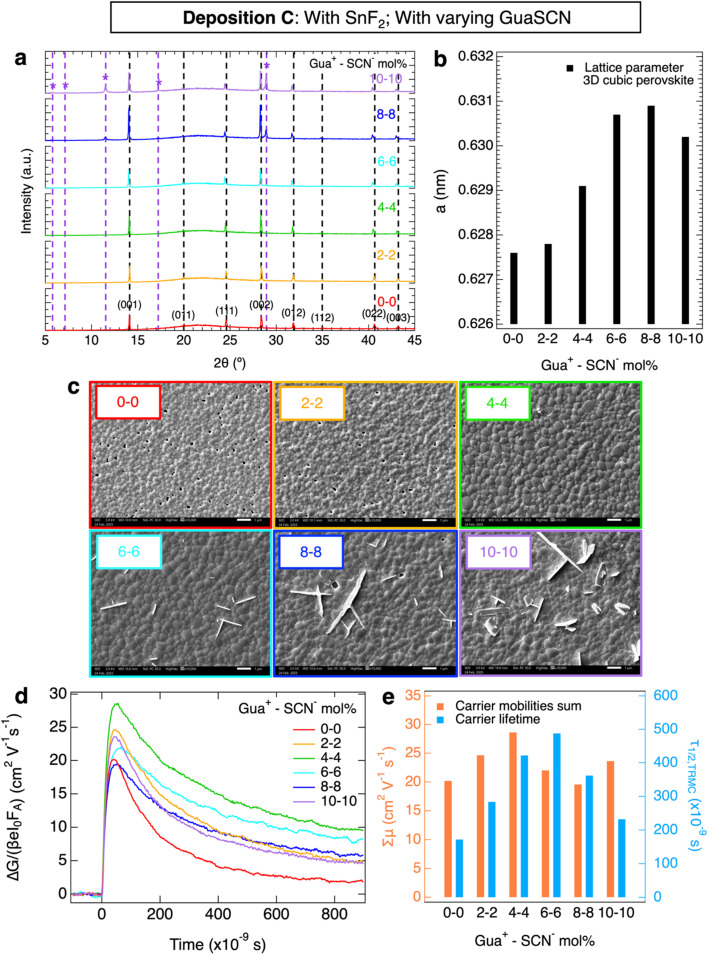
Effect of varying GuaSCN mol% and 10 mol% SnF_2_ on the microstructure and charge carrier transport properties of perovskite thin films. In (a), the XRD shows peaks corresponding to a 3D cubic perovskite phase (black dashed lines and Miller indices) and to additional Gua-based 2D perovskite phases for the addition of ≥6 mol% GuaSCN (purple dashed lines and (*) markers). In (b), the lattice parameter, *a*, of the 3D cubic perovskite phase is also shown. In (c), the SEM images were taken at 100 00× magnification. Regarding the charge carrier properties, the (d) TRMC photoconductivity transients obtained with laser excitations *λ* = 800 nm at the same intensity and (e) carrier mobilities sum, Σ*µ*, and lifetimes, *τ*_1/2_, are shown.

Next, we studied the perovskite layers from deposition C all with GuaSCN varying from 0 mol% to 10 mol% w.r.t. (CsI + FAI) and 10 mol% SnF_2_ w.r.t. SnI_2_ in Fig. 3. The XRD patterns in Fig. 3a show the 3D cubic perovskite phase for GuaSCN additions ≤ 4 mol%. As visible in Fig. 3b, on increasing the GuaSCN content, the XRD peaks shift gradually to lower angles (in detail in Fig. S3a–c), *i.e.* the lattice parameter of the cubic phase becomes larger (*a* = 0.628 nm → *a* = 0.631 nm) due to the incorporation of Gua^+^ in the cubic perovskite structure, but stops expanding for samples with ≥6 mol% GuaSCN. Hence, we deduce that for ≥6 mol% GuaSCN, the excess Gua^+^ cannot be incorporated anymore into the 3D cubic perovskite crystal and forms additional phase(s), most likely Gua-based low-dimensional phases, as reported in the literature.^21,22,29,30^ In line with it, additional XRD peaks appear for ≥6 mol% GuaSCN, which we attribute to these crystal phases and will be discussed below. Moreover, the crystallinity of the 3D cubic perovskite phase hardly changes with varying GuaSCN addition, as shown by the FWHM of the XRD peaks (see also Fig. S3d).

Regarding the microstructure, the grains as observed in the SEM images, shown in Fig. 3c, S15 and S16, become larger for increasing concentrations up to 4 mol% GuaSCN, creating films with no obvious pinholes. For ≥6 mol% <*D*> stops increasing and large platelet-like crystals appear alongside the polycrystalline films, oriented nearly perpendicular to the substrate as shown in the SEM images. We speculate that the 3D cubic perovskite phase forms the polycrystalline layer, while the additional low-dimensional Gua-based phases correspond to the platelet-shaped crystals.

Then, we carried out an elemental analysis of the perovskite films using XPS on the samples with 0, 4 and 10 mol% GuaSCN of deposition C. To quantify the maximum incorporated amount of Gua^+^ in the bulk 3D cubic perovskite structure, we focused on the layer with 4 mol% GuaSCN given that Gua^+^ starts to form 2D crystal phases for ≥6 mol% GuaSCN. From the XPS scans on C 1s and N 1s for this film in Fig. S26e and f, no clear XPS peaks that can reliably confirm the presence of Gua^+^. Therefore, the fraction of incorporated Gua^+^ in the bulk 3D perovskite is limited, considering the content < 1 at% C/N assigned to Gua^+^, *i.e.* below the XPS detection limit. With respect to the possible incorporation of SCN^−^, analysis of the S 2p, C 1s and N 1s orbitals of the XPS scans for all GuaSCN concentrations in Fig. S26 provides no evident signs of SCN^−^. Hence, we conclude that SCN^−^ is not built-in in the perovskite films. Although the absence of SCN^−^ in the crystallized films is reported in other works, allegedly due to the evolution of crystallization-assisting gaseous compounds during the perovskite annealing,^23,27,33,35^ we think that the majority of SCN^−^ is lost with the excess solution during spin-coating.

We also analyzed the optical properties of the layers by UV-Vis shown in Fig. S5c. The maximum absorption in the 700 to 900 nm region is slightly reduced for films with ≥8 mol% GuaSCN, most likely due to a decrease in the thickness of the 3D cubic phase. Moreover, we studied the conductivity of these films. SSMC measurements (Fig. S6c) show that all layers are nearly intrinsic with *σ*_dark_ ≤ 1 S m^−1^, as expected since they all contain 10 mol% SnF_2_. In addition, TRMC traces recorded at the same intensity and wavelength are shown in Fig. 3d, while the corresponding charge carrier transport properties are plotted in Fig. 3e. We note that the mobility–lifetime product increases with higher GuaSCN concentrations, reaching the optimum for 4 mol% GuaSCN, which presents a *L*_D_ of ∼4 µm (Fig. S10c). For ≥6 mol% GuaSCN, the mobility–lifetime product decreases again.

To identify the low-dimensional phases formed on adding ≥6 mol% GuaSCN, we thoroughly studied the XRD patterns of films with 10 mol% GuaSCN. Since we know by comparing Fig. 1 and 2 that the addition of SnF_2_ leads to lower intensity of the XRD peaks due to the limited grain growth and texture, we also deposited a perovskite film with 10 mol% GuaSCN and no SnF_2_ addition, whose structural, microstructural and opto-electronic analyses are shown in Fig. S27 in comparison with a reference layer without additives. Indeed, the XRD pattern of this layer in Fig. S27a presents extremely high-intensity XRD peaks and strongly enlarged grains. As a result, the XRD peaks attributed to the low-dimensional phases become clearly visible. The XRD patterns of both layers with 10 mol% GuaSCN, with or without SnF_2_, were compared to simulated reference patterns comprising impurities (Fig. S20), 1D (Fig. S21) and 2D Gua^+^-containing perovskite phases (Fig. S22), reported in the literature and databases.^29,37–46^

We attributed the XRD peaks to specific sets of crystal planes belonging to the 3D cubic perovskite phase and the remaining peaks to Gua-based 2D perovskite phases (Fig. S23, Tables S1 and S2), which we identified as Gua_*x*_FA_2−*x*_Sn_*y*_Pb_1−*y*_I_4_ (*n* = 1) and Gua_*x*_FA_3−*x*_Sn_*y*_Pb_2−*y*_I_7_ (*n* = 2), where *n* is the number of inorganic octahedra layers. We highlight that the appearance of the large platelet-shaped crystals, *i.e.* bidimensional microstructures growing along two preferential directions, observed in the SEM images (Fig. 3c) corresponds to the emergence of the layered Gua-based 2D crystal phases in the XRD patterns for ≥6 mol% GuaSCN addition. Unfortunately, back-scattered electron SEM images (Fig. S18) and spatially resolved EDX (Fig. S19) did not reveal consistent differences between these platelet-shaped crystals and the polycrystalline film. However, this can be explained by the limited micrometer-sized resolution of both types of analyses.

In the literature, the formation of Gua-based 2D perovskite crystal phases is considered beneficial for the carrier transport in the perovskite films, due to defect passivation at GBs, faster charge extraction at the interfaces with the 3D cubic perovskite and the formation of a protective barrier against oxygen.^21,22^ However, *n* also appears to play an important role. Low mobilities and short charge carrier lifetimes have been reported for 2D perovskite phases with *n* = 1, such as Gua_2_PbI_4_.^22^ In contrast, improved carrier transport has been observed in the literature for PEA- and Gua-containing 2D perovskites with *n* = 2.^22^ In our work, we notice a positive effect on the opto-electronic properties of Sn–Pb perovskite films as long as the concentration of added Gua^+^ is ≤4 mol%. In this way, part of Gua^+^ is incorporated in the 3D cubic perovskite improving the mobility–lifetime product. Contrarily, if Gua-based 2D phases appear for ≥6 mol% GuaSCN, the carrier transport in the 3D perovskite layers declines, as indicated by the observed decrease in the mobility–lifetime product. Although the addition of GuaSCN always improves the carrier transport in comparison to the reference layer, our results indicate that the formation of Gua-based 2D phases is not essential to enhance the opto-electronic properties of the 3D perovskite films. In fact, the accumulation of these 2D phases at high GuaSCN concentrations disrupts the morphology of the polycrystalline perovskite films by forming large platelet-like crystals, which might hamper the carrier transport at the interfaces in a full solar cell. Hence, we suggest that adding ≥6 mol% GuaSCN in mixed Sn–Pb perovskites is not necessary and even potentially harmful for the corresponding devices.

### Effect of Gua^+^

Considering the results of depositions A and B, we can conclude that the addition of only Gua^+^ positively affects the carrier transport, but only when SnF_2_ is present to counteract tin oxidation and doping. This implies that Gua^+^ is not scavenging oxidized Sn^2+^ species during the spin-coating and crystallization process. In deposition B, with 4 mol% Gua^+^ and SnF_2_, we observe clear defect passivation since the TRMC traces from Fig. 2e at low intensities exhibit substantially longer lifetimes than the corresponding layer without Gua^+^. The mechanism is not fully clear yet. It may be due to direct passivation by Gua^+^ ions *via* the amino groups (–NH_2_) or indirect passivation *via* hydrogen bonds (N–H⋯I) of crystal defects such as under-coordinated ions.^29–31,58,59^ Previous claims that attribute defect passivation to the formation of low-dimensional phases are not in agreement with our observations.^21,22^

From the XRD measurements on depositions A, B and C we observe that a small fraction of Gua^+^ is incorporated in the 3D perovskite, very likely in the A-sites. This might be related to the increased number of hydrogen bonding interactions between the amino groups and I^−^ ions formed by Gua^+^ (6) compared to FA^+^ (4) in the perovskite structure,^29,58^ which shorten the H–I distance and anchor the I^−^ ions.^30^ The resulting stiffer crystal and electronic band structure are expected to increase the carrier scattering time and thereby the mobilities.^26,30,60^

### Effect of SCN^−^

From our observations on depositions A and B, we conclude that addition of SCN^−^ reduces doping and defect formation during spin-coating leading to Sn–Pb perovskites with reduced doping, comparable to the function of SnF_2_. However, in combination with SnF_2_, a clear improvement of the charge carrier lifetimes was found, hinting towards a complimentary passivating effect. To verify if indeed SCN^−^ scavenges Sn^4+^ from the spin coating solution, we dissolved SnI_4_ in a solvent mixture of DMF and DMSO with a volumetric ratio of 4 : 1. The absorbance spectrum in Fig. S4 shows two absorption features, namely a peak at ∼332 nm and another shallow peak around ∼420 nm. Addition of Pb(SCN)_2_ to the SnI_4_ solution yields an immediate color change from yellow to transparent. In the absorption spectrum, two new absorbance peaks at ∼283 nm and a shallow peak at ∼331 nm are observed. We conclude that the original SnI_4_, likely coordinated by DMSO in the form of SnI_4_·(DMSO)_2_ complexes,^61^ forms a different complex coordinated by SCN^−^.^62^ In the spin-coating solvent we expect that Sn^4+^ forms similar complexes leading to its removal, which results in reduced doping and crystal defects in the perovskite layers in line with previous reports.^20,27,34^ Hence, we inferred that SCN^−^ and SnF_2_ have a similar effect against tin oxidation, but the mechanism is rather different. In fact, SCN^−^ suppresses tin oxidation *via* coordination with Sn^4+^ in the spin-coating solution, preventing the incorporation within the perovskite layer. Instead, SnF_2_ is involved in a ligand exchange reaction with the Sn^4+^ in solution supplying new Sn^2+^.^16^

Moreover, as mentioned in the literature,^27,28^ SCN^−^ ions can form an S-bonded coordination with Sn^2+^ ions by donating a lone pair electron to the empty p orbitals of the metal ion. We speculate that this coordination between SCN^−^ and Sn^2+^ slows down perovskite formations,^27^ assisting the film crystallization, favoring the grain growth and reducing the defect density. This leads to the improved transport properties found on adding SCN^−^ in the presence of SnF_2_. Since even at 10 mol% GuaSCN no signs of SCN^−^ could be found, we deduce that SCN^−^ is lost during the perovskite synthesis and/or annealing.

### Effect of GuaSCN

**Scheme 2:**
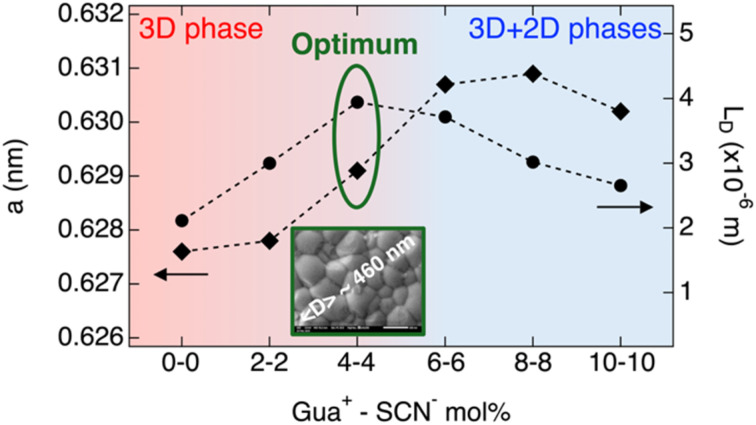
Lattice parameter of the cubic 3D perovskite, *a*, and carrier diffusion length, *L*_D_, as a function of the concentration of Gua^+^ and SCN^−^ ions by GuaSCN addition in the perovskite precursor solution. The optimum GuaSCN additive concentration in terms of structural, microstructural and carrier transport properties of the mixed Sn–Pb perovskite film is marked (dark green). The inset shows a top-view SEM image at 50 000× magnification of the optimized perovskite film, where the corresponding average grain size, <*D*>, is also indicated.

Finally, we want to address the effect of the combined additives. Interestingly, the dependence of *L*_D_ on the GuaSCN concentration is interconnected to the changes in the structural and microstructural properties, as summarized in Scheme 2. The largest *L*_D_ ∼ 4 µm is achieved when the perovskite film consists of a 3D perovskite incorporating a small amount of Gua^+^, as shown by the slightly increased *a* of the cubic phase and no detectable 2D phases. Once 2D phases begin to form, *L*_D_ decreases and continues to decline as their content increases. Moreover, we concluded that while the addition of either Gua^+^ and/or SCN^−^ in combination with 10 mol% SnF_2_ similarly improves the mobility–lifetime product, only the combined effect of Gua^+^ and SCN^−^ until a maximum addition of 4 mol% GuaSCN results in a synergistic effect comprising the formation of large grains and pinhole-free perovskite layers. Hence, we recommend the combination of both additives for producing high-quality Sn–Pb perovskite thin films, promising for implementation in full solar cells. In the future, further studies are needed to gain a deeper understanding of the mechanism underlying the improved film microstructure resulting from the combined addition of Gua^+^ and SCN^−^, particularly focusing on the chemistry in the perovskite precursor solution and the crystallization dynamics of the films.

**Fig. 4:**
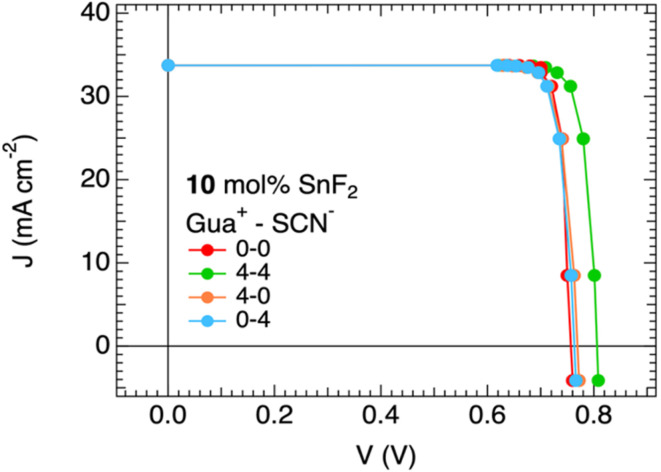
Separate and combined contributions of Gua^+^ and/or SCN^−^ ions to the pseudo-*J*–*V* curves of mixed Sn–Pb perovskite films from deposition B, comparing the reference film to layers with 4 mol% Gua^+^ and/or SCN^−^, all with 10 mol% SnF_2_.

To corroborate our clams regarding possible applications in solar cells, we performed microwave-based quasi-Fermi level splitting (QFLS) measurements on the films from deposition B, following the method described in previous works.^47,63^ The QFLS represents the maximum achievable voltage obtainable in the absorber layer. From the intensity-dependent QFLS measurements and following reported procedures,^48,49^ we derived the pseudo-*J*–*V* curves. The resulting pseudo-*J*–*V* curves are shown in Fig. 4.

We noticed that the *J*–*V* curves for the reference film and the films with only Gua^+^ or only SCN^−^ are alike. In contrast, the pseudo-open circuit voltage, *pV*_OC_, of the layer with GuaSCN is significantly increased by 50 mV. All the pseudo-fill factors are high and all very close to 0.9 (see the pseudo-*J*–*V* parameters in Tables S3–S6), which is explained by the absence of any series resistance in the pseudo-*J*–*V* curves. In line with our findings, the mixed Sn–Pb perovskite films with 4 mol% GuaSCN in combination with SnF_2_ appear as absorbers with the largest potential for implementation in efficient solar cells. These results further demonstrate a synergistic interaction between Gua^+^ and SCN^−^ which enhances the optoelectronic properties of the mixed Sn–Pb perovskite layer when these ions are used together rather than individually.

## Conclusions

To study the separate and combined effects of Gua^+^ and/or SCN^−^ ions on mixed Sn–Pb perovskites, we added small volumes of solutions of GuaI, Pb(SCN)_2_ and GuaSCN, to a Cs_0.25_FA_0.75_Sn_0.5_Pb_0.5_I_3_ perovskite parent solution in the absence or in the presence of SnF_2_ and used these mixtures to spin-coat thin films. We acknowledge that even small deviations (0.5–1%) from the ideal perovskite precursor stoichiometry can alter the relative device performance. However, to avoid making the systems even more complex by choosing foreign cations, we selected counterions already present in the precursor solution. For each deposition, we investigated the structural (XRD), microstructural (SEM), optical (UV-Vis), charge carrier transport in the dark and under illumination (SSMC and TRMC) and compositional properties (XPS). By comparing the perovskite films, we elucidated the function of each additive, crucial for tuning the perovskite properties and obtaining absorber layers for highly efficient Sn–Pb perovskite solar cells.

Gua^+^ is partially incorporated in the 3D cubic perovskite crystal structure as shown by the lattice expansion, although the fraction of incorporated Gua^+^ in the bulk 3D perovskite is <1 at%. Without SnF_2_, the addition of only Gua^+^ does not prevent tin oxidation and doping, resulting in unsatisfactory carrier transport properties (both low carrier mobilities and lifetimes) and making it useless for Sn–Pb perovskite absorber layers in a solar cell. However, when combined with SnF_2_ which counteracts tin oxidation, the addition of Gua^+^ increases both the mobilities and lifetimes likely due to the increased number of hydrogen bonding interactions, reduced H–I distance and defect passivation in the perovskite crystal. Thus, Gua^+^ is beneficial in Sn–Pb perovskites only when paired with SnF_2_.

SCN^−^ suppresses oxidation, passivates crystal defects and always improves the mobility–lifetime product. We demonstrated that the original SnI_4_, likely coordinated by DMSO in the form of SnI_4_·(DMSO)_2_ complexes, forms a different complex coordinated by SCN^−^. The scavenging of Sn^4+^ in the spin-coating solution and pile-up of SnO_*x*_ at the film surface explain the function of SCN^−^ ions in suppressing tin oxidation, doping and associated defects and improving the carrier transport properties, which is fundamental when SnF_2_ is absent. However, it seems that SCN^−^ does not remain in the crystallized and annealed perovskite films.

The films with 4 mol% GuaSCN and 10 mol% SnF_2_ yield the maximum mobility–lifetime product and carrier diffusion length of ∼4 µm. However, when the GuaSCN concentration is ≥ 6 mol%, additional Gua-containing 2D perovskite phases *i.e.* Gua_*x*_FA_2−*x*_Sn_*y*_Pb_1−*y*_I_4_ and Gua_*x*_FA_3−*x*_Sn_*y*_Pb_2−*y*_I_7_ appear. Importantly, the accumulation of these 2D phases does not lead to improved charge carrier mobilities or lifetimes. We conclude that only the synergistic effect of Gua^+^ and SCN^−^ until a maximum addition of 4 mol% and 10 mol% SnF_2_ produces large grains and pinhole-free perovskite thin films with superior charge carrier transport properties, which is promising for implementation in full solar cells.

## Conflicts of interest

There are no conflicts to declare.

## Supplementary Material

TA-014-D5TA08016A-s001

## Data Availability

Supplementary information (SI): XRD, XPS, UV-Vis-NIR spectroscopy, SSMC and TRMC results, as well as the SEM images. See DOI: https://doi.org/10.1039/d5ta08016a.
