# Supplementary material for: Separate and combined effects of Gua+ and SCN− ions on charge carrier dynamics in mixed Sn–Pb perovskites
Source: J Mater Chem A Mater. 2026 Jan 26;14(14):8294–306. doi: 10.1039/d5ta08016a (PMC12834201; doi:10.1039/d5ta08016a)
Supplement: TA-014-D5TA08016A-s001 [file TA-014-D5TA08016A-s001.pdf]

Supplementary Information (SI) for Journal of Materials Chemistry A.
This journal is © The Royal Society of Chemistry 2026

Separate and Combined Effect of Gua^+ and SCN^- Ions on the Charge Carrier Dynamics in Mixed Sn-Pb Perovskites

Authors

Jasmeen Nespoli[†], Maartje J. van der Meer[†], Lara M. van der Poll[†], Xiaohui Liu[†], Tom J. Savenije[*,†]

Affiliations

[†]*Department of Chemical Engineering, Faculty of Applied Sciences, Delft University of Technology, 2629 HZ Delft, The Netherlands*

Corresponding Author

Tom J. Savenije - E-mail: T.J.Savenije@tudelft.nl

Supporting information (SI)

X-Ray Diffraction (XRD) – Crystal structure and XRD peaks full-width half maximum (FWHM)

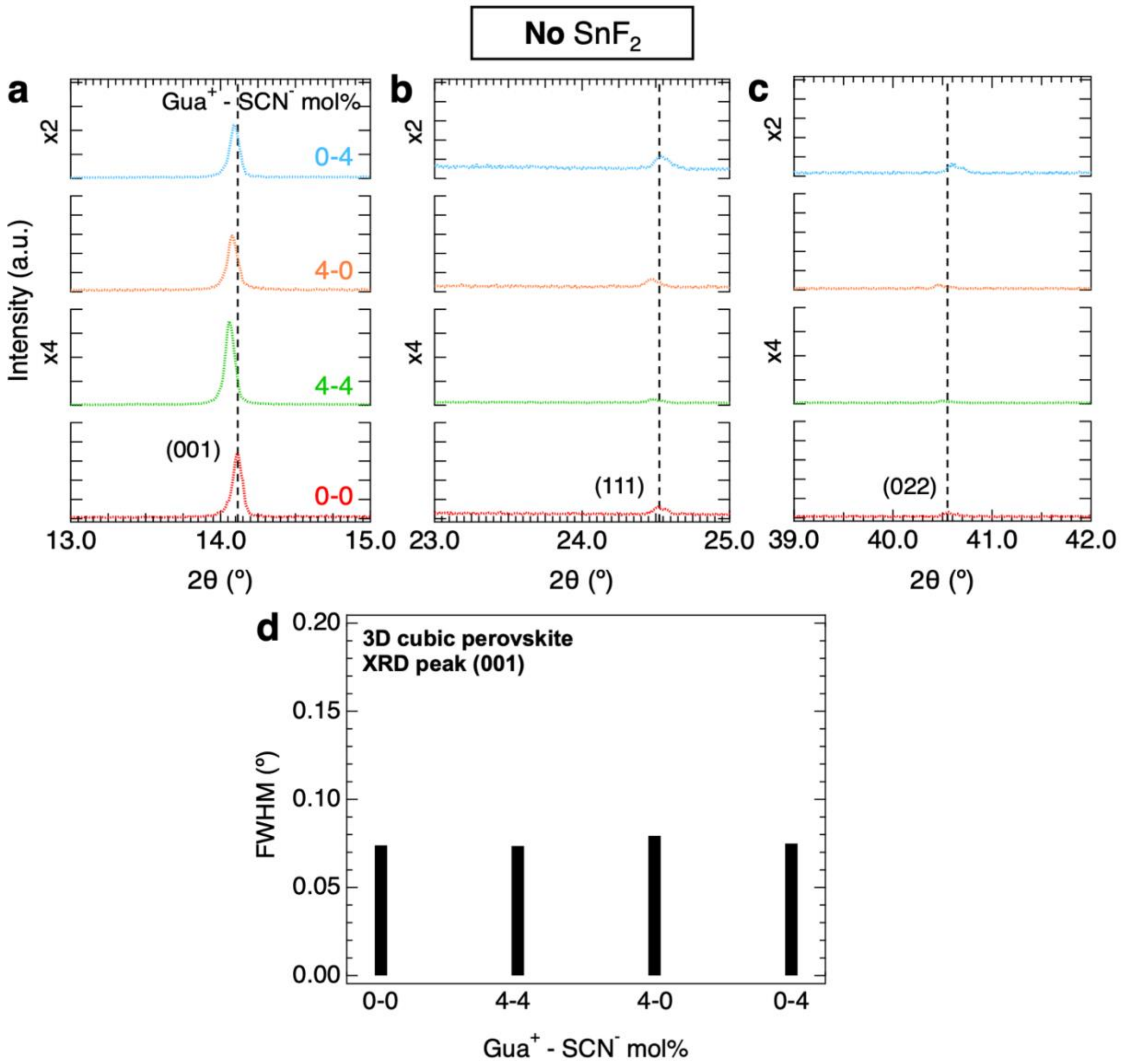

Figure S1: Effect of Gua^+ and/or SCN^- ions, by addition of 0 and 4 mol% Gua^+ and SCN^- ions and 0 mol% SnF_2, showing the XRD patterns and analysis of the peaks (001), (111) and (022) of the 3D cubic perovskite phase (black dashed lines and Miller indices). To facilitate the comparison, note that the XRD intensity axes were rescaled by factors of ×2 and ×4 for the layers containing 4 mol% SCN^- and 4 mol% GuaSCN, respectively. Full-width half maximum (FWHM) of the (001) peak of the 3D cubic perovskite phase as a function of varying Gua^+ and SCN^- mol%.

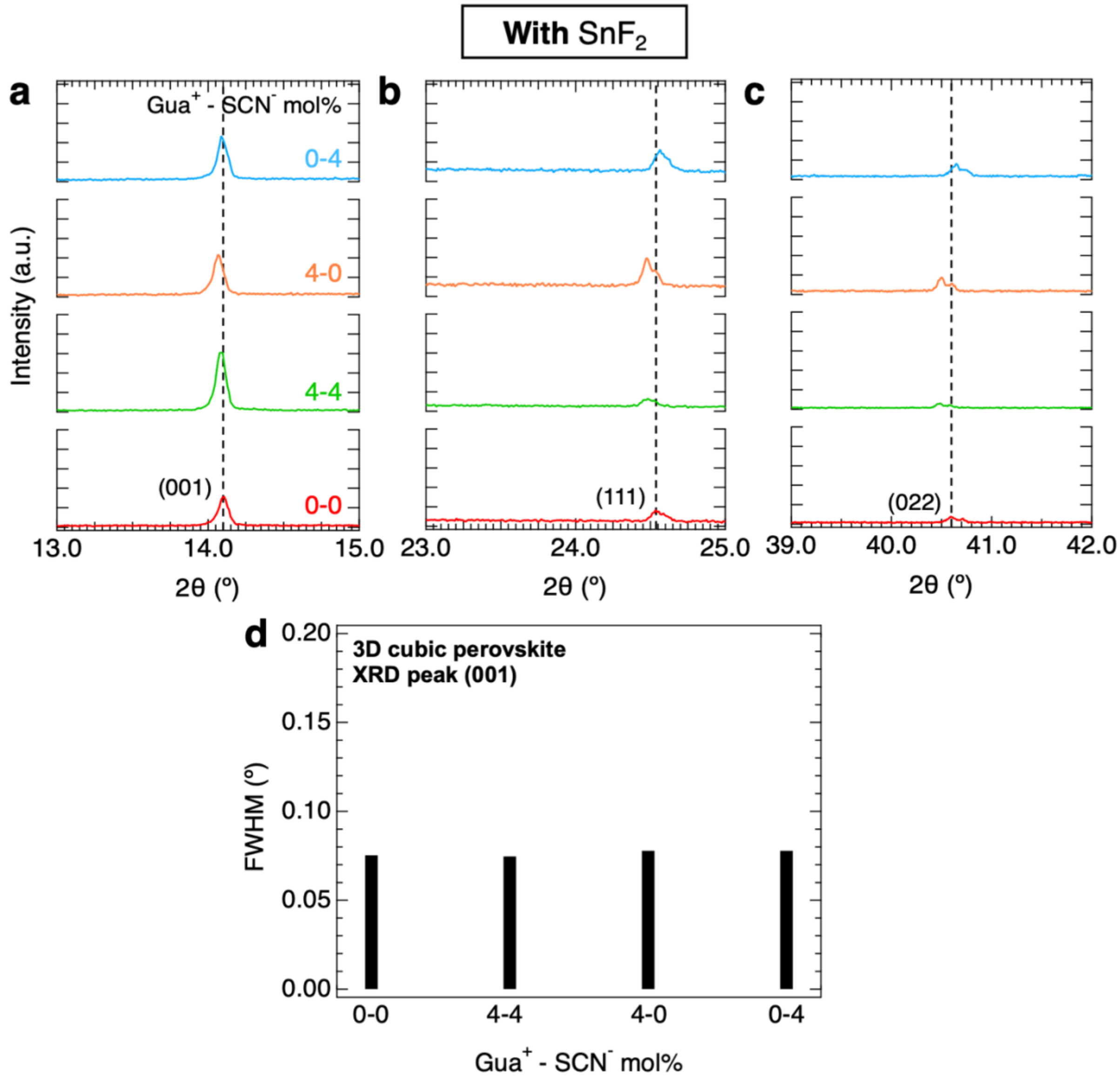

Figure S2: Separate contributions of Gua^+ and SCN^- ions on the crystal structure properties by addition of 0 and 4 mol% Gua^+ and SCN^- ions and 10 mol% SnF_2, showing the XRD patterns and analysis of the peaks (001), (111) and (022) of the 3D cubic perovskite phase (black dashed lines and Miller indices). Full-width half maximum (FWHM) of the (001) peak of the 3D cubic perovskite phase as a function of varying Gua^+ and SCN^- mol%.

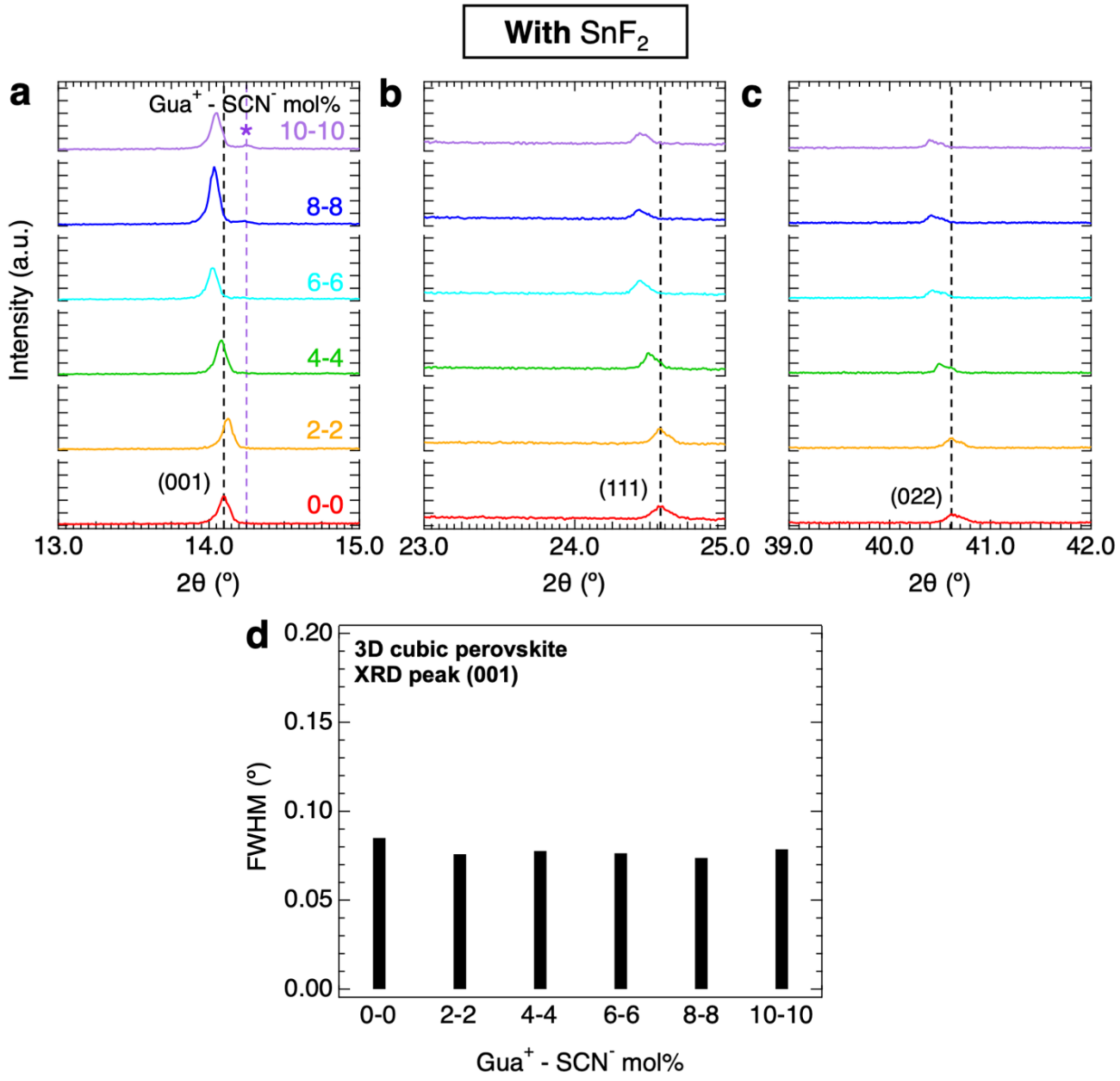

Figure S3: Separate contributions of Gua^+ and SCN^- ions on the crystal structure properties by addition of 0 and 4 mol% Gua^+ and SCN^- ions and 10 mol% SnF_2, showing the XRD patterns and analysis of the peaks (001), (111) and (022) of the 3D cubic perovskite phase (black dashed lines and Miller indices). Full-width half-maximum (FWHM) of the (001) peak of the 3D cubic perovskite phase as a function of varying Gua^+ and SCN^- mol%.

UV-Vis-NIR Spectroscopy (UV-Vis) – Absorption spectra

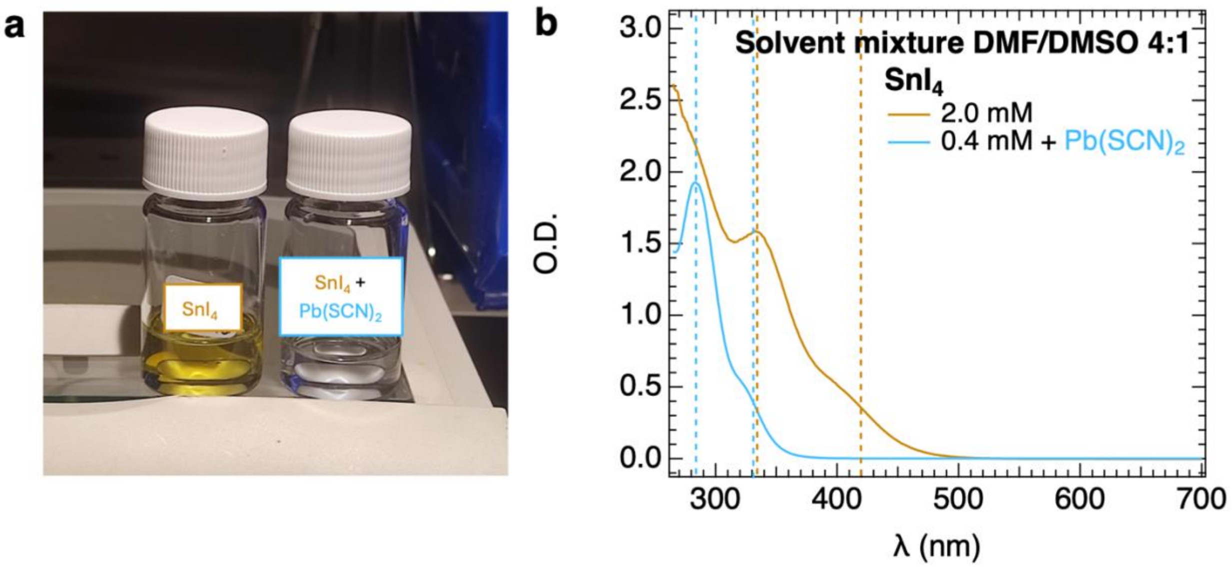

Figure S4: (a) Photographs and (b) optical density, *O.D.*, spectra of SnI_4 solution (0.2 mM) in a mixture of DMF and DMSO, without (in dark yellow) and with added $Pb(SCN)_2$ (in light blue), where the absorption peaks are also shown (dashed lines). For the SnI_4 + $Pb(SCN)_2$ solution, we added 7 mg of $Pb(SCN)_2$ to 4 mL of SnI_4 solution, we stirred it for 1 h and diluted it to 0.4 mM.

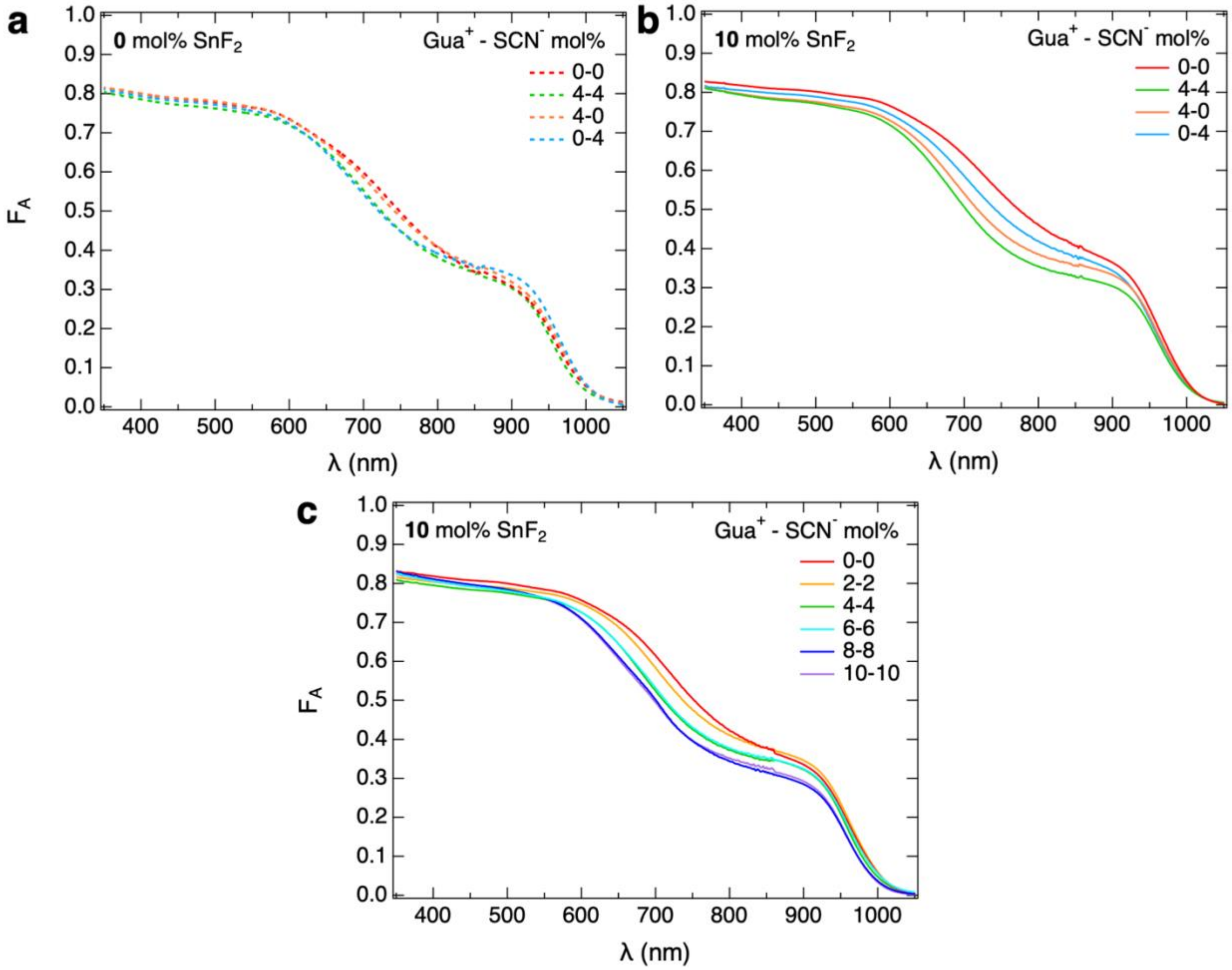

Figure S5: Absorptance spectra of perovskite thin films with (a-b) 0 and 4 mol% Gua^+ and SCN^- ions. In details, (a) and (b) show films respectively with 0 and 10 mol% SnF_2 addition. (c) Absorptance spectra of perovskite thin films with varying GuaSCN mol% and 10 mol% SnF_2 addition.

Steady State Microwave Conductance (SSMC) – Dark conductivity

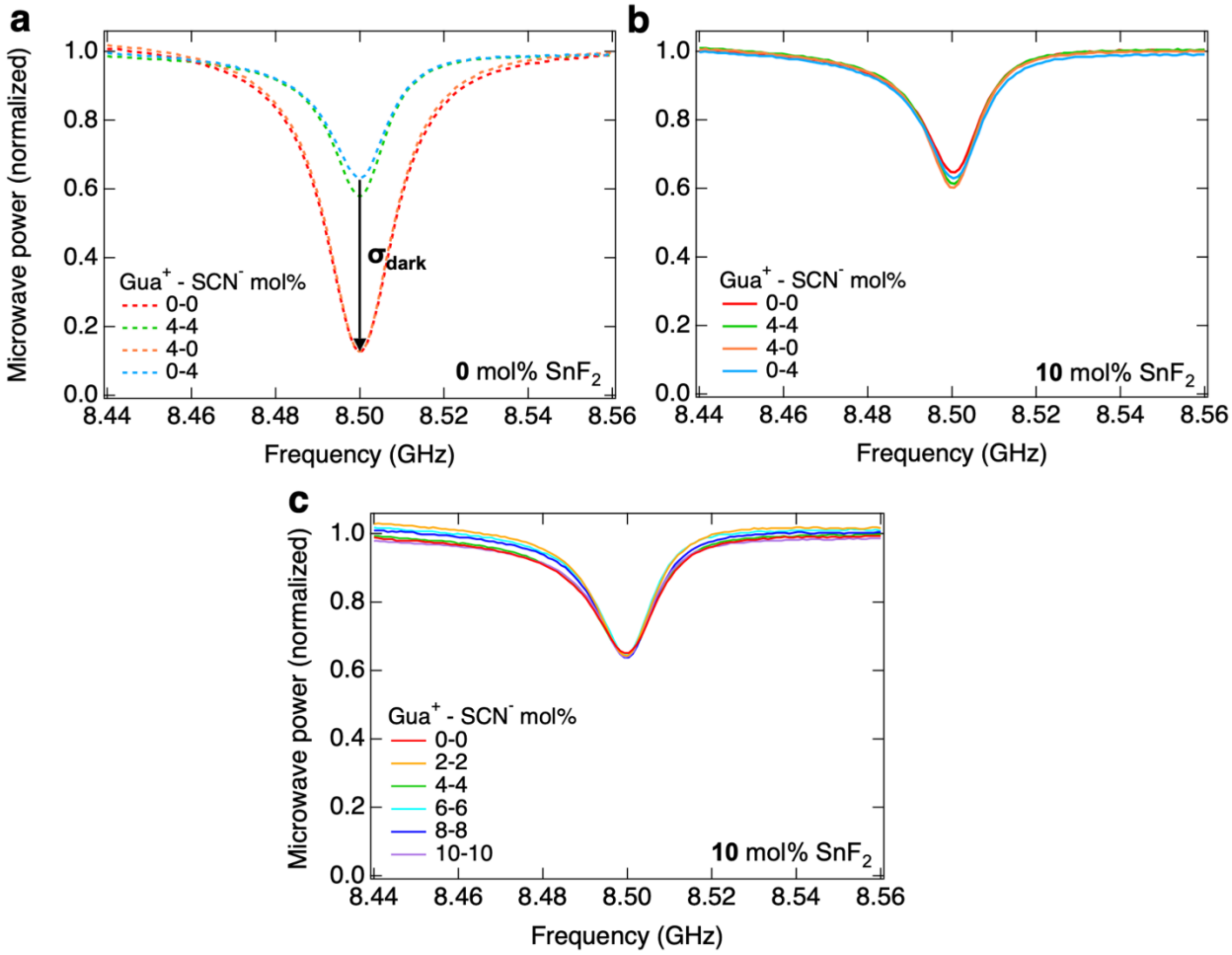

Figure S6: SSMC frequency scans of perovskite thin films with (a-b) 0 and 4 mol% Gua^+ and SCN^- ions. In details, (a) and (b) show films respectively with 0 and 10 mol% SnF_2 addition. (c) SSMC frequency scans of perovskite thin films with varying GuaSCN mol% and 10 mol% SnF_2 addition.

Time-resolved Microwave Conductivity (TRMC) - Photogenerated charge carrier Dynamics and Diffusion Lengths

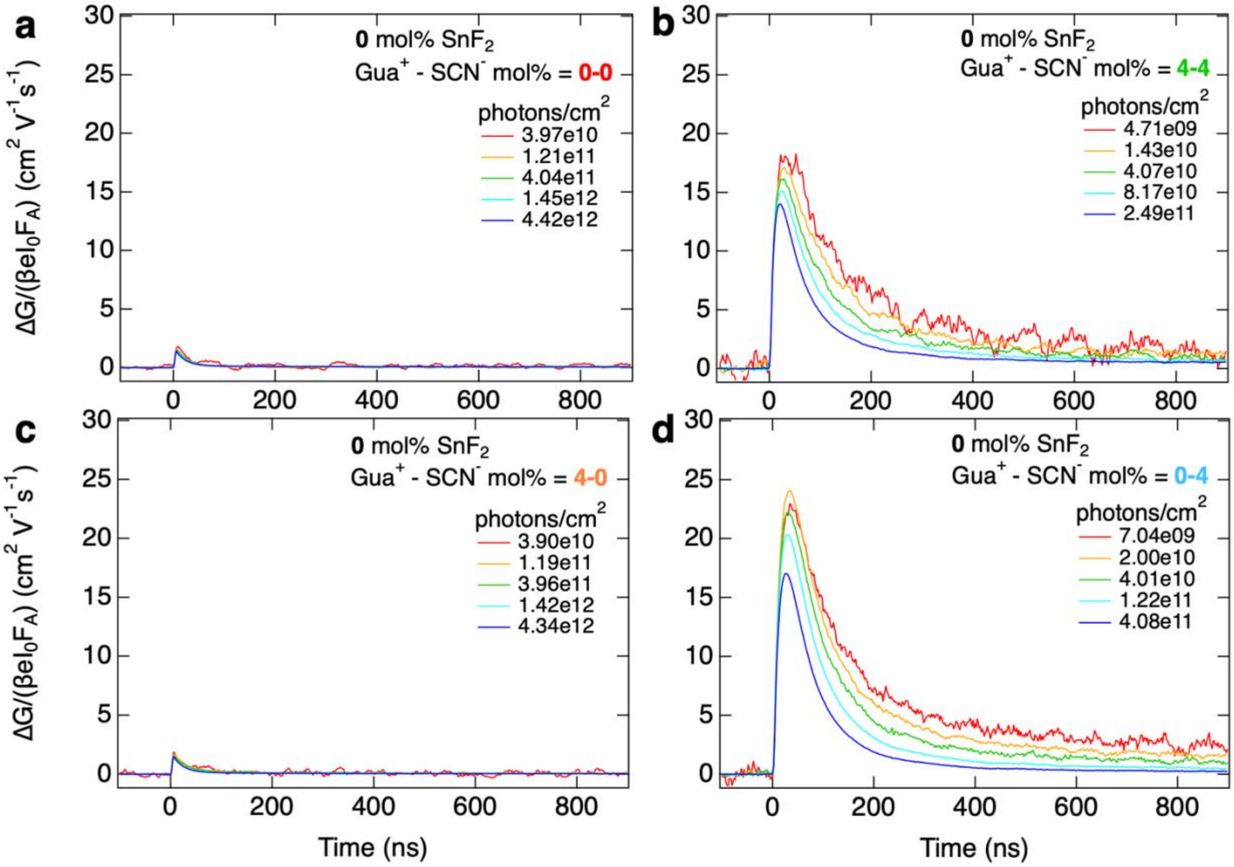

Figure S7: Intensity-dependent TRMC traces of perovskite thin films with addition of 0 or 4 mol% Gua^+ and SCN^- ions and 0 mol% SnF_2 addition. All the TRMC measurements were performed at the same excitation wavelength (λ = 800 nm) and corrected for the absorbed fraction of light in **Figure S5a** at such wavelength.

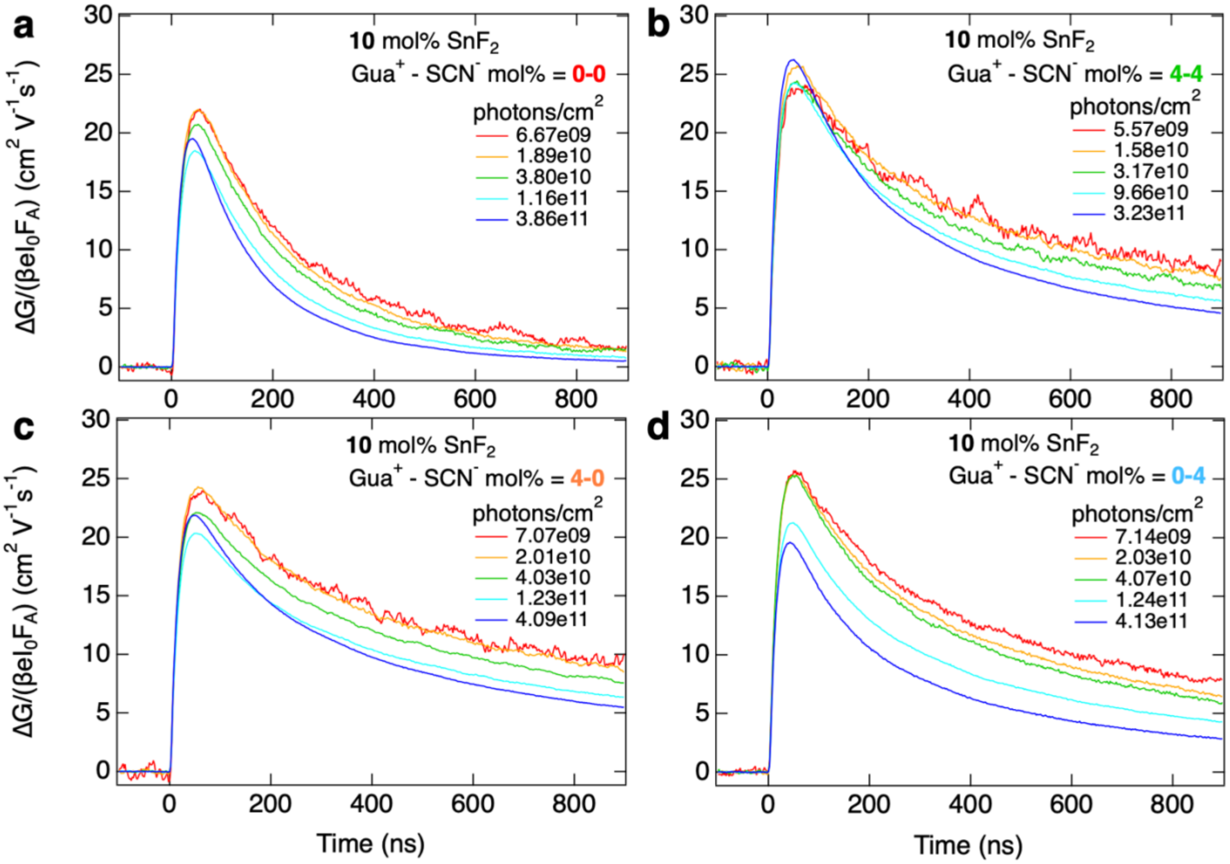

Figure S8: Intensity-dependent TRMC traces of perovskite thin films with addition of 0 or 4 mol% Gua^+ and SCN^- ions and 10 mol% SnF_2 addition. All the TRMC measurements were performed at the same excitation wavelength (λ = 800 nm) and corrected for the absorbed fraction of light in **Figure S5b** at such wavelength.

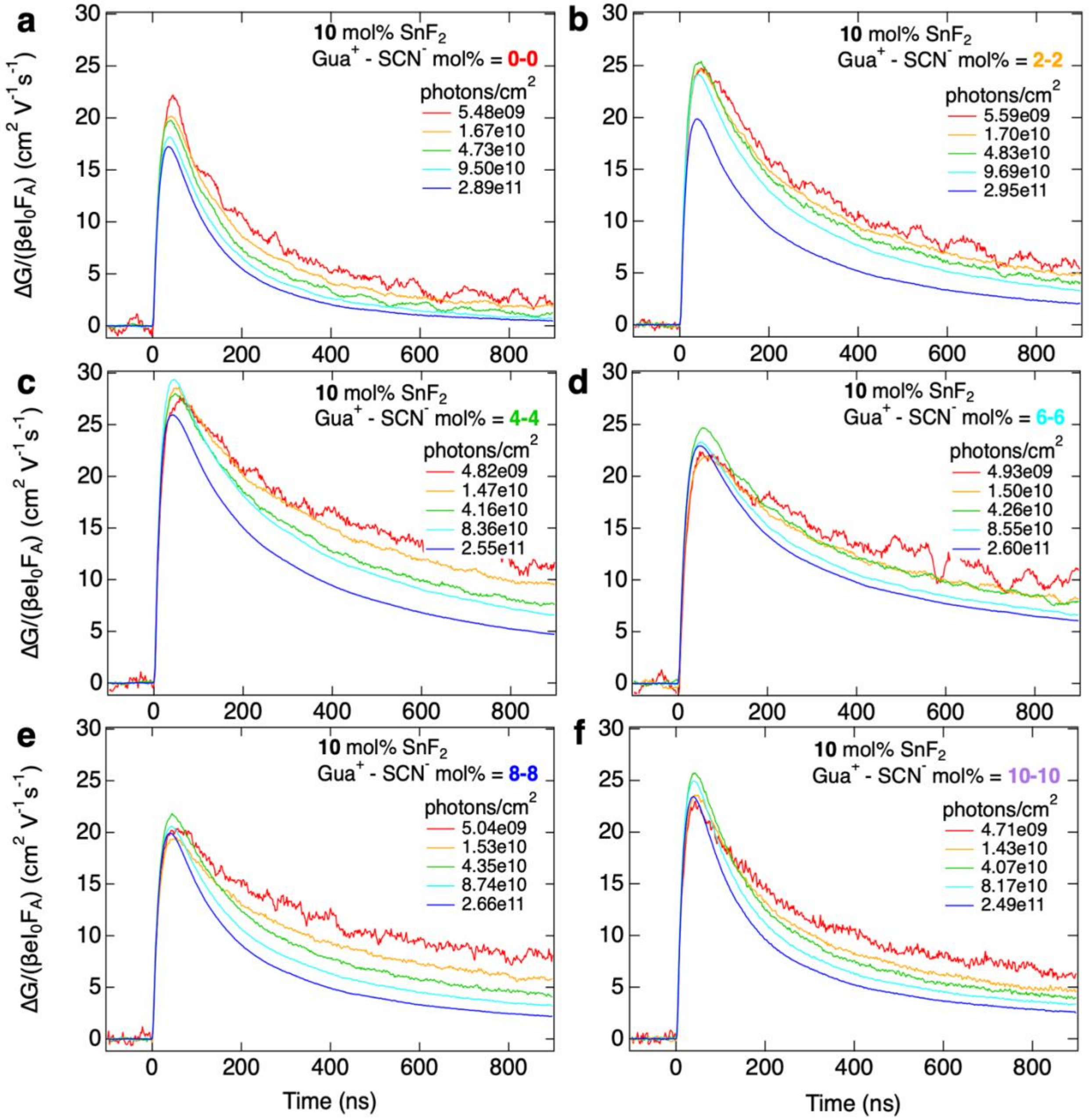

Figure S9: Intensity-dependent TRMC traces of perovskite thin films with varying GuaSCN mol% and 10 mol% SnF_2 addition. All the TRMC measurements were performed at the same excitation wavelength (λ = 800 nm) and corrected for the absorbed fraction of light in **Figure S5c** at such wavelength.

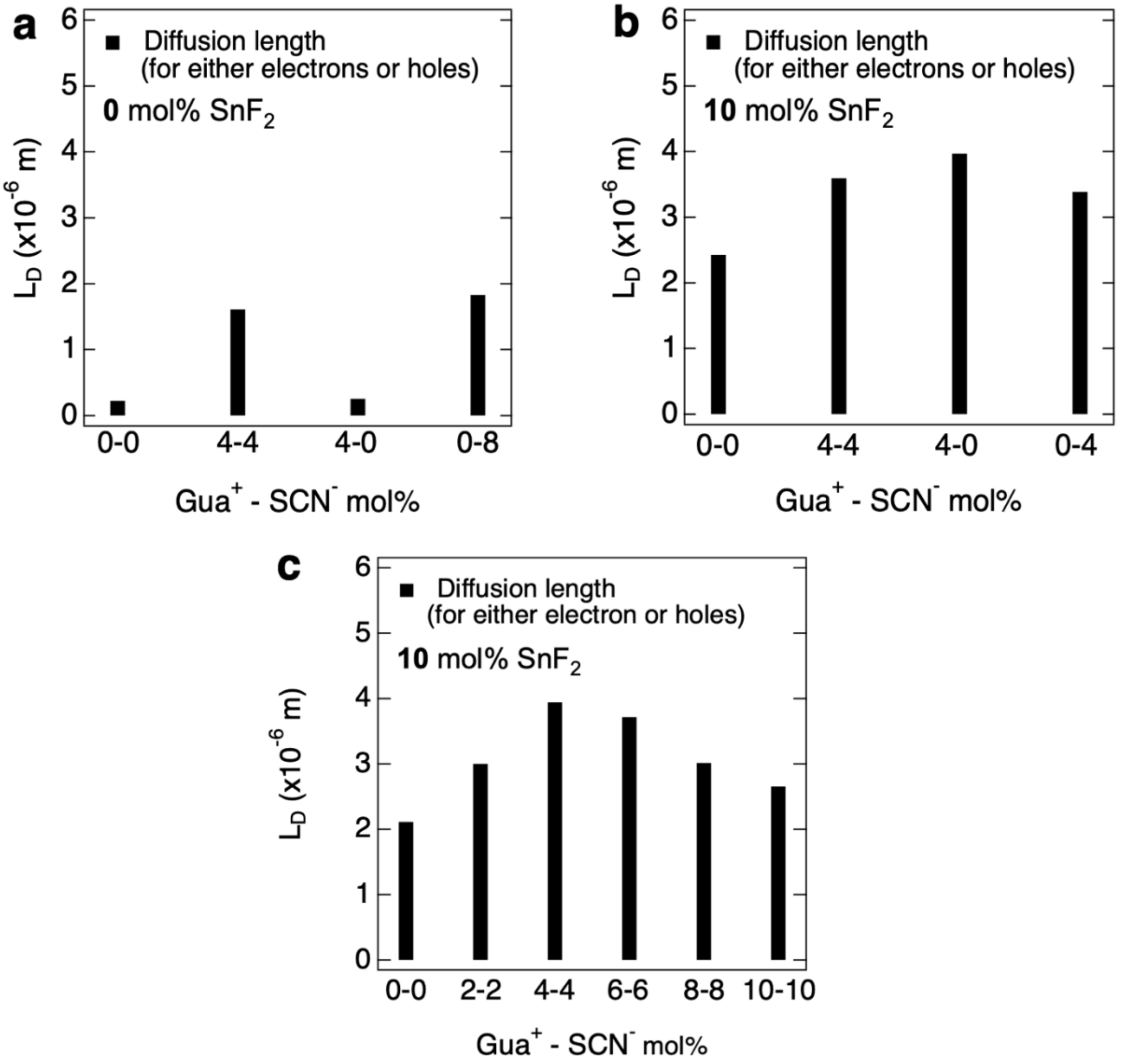

Figure S10: Carrier diffusion length, L_D, of perovskite thin films with (a-b) 0 or 4 mol% Gua^+ and SCN^- ions. In details, (a) and (b) show films respectively with 0 and 10 mol% SnF_2 addition. (c) L_D of perovskite thin films with varying GuaSCN mol% and 10 mol % SnF_2 addition.

Scanning Electron Microscopy (SEM) and Electron Dispersive X-ray (EDX) analysis – Microstructure, average grain size and elemental maps

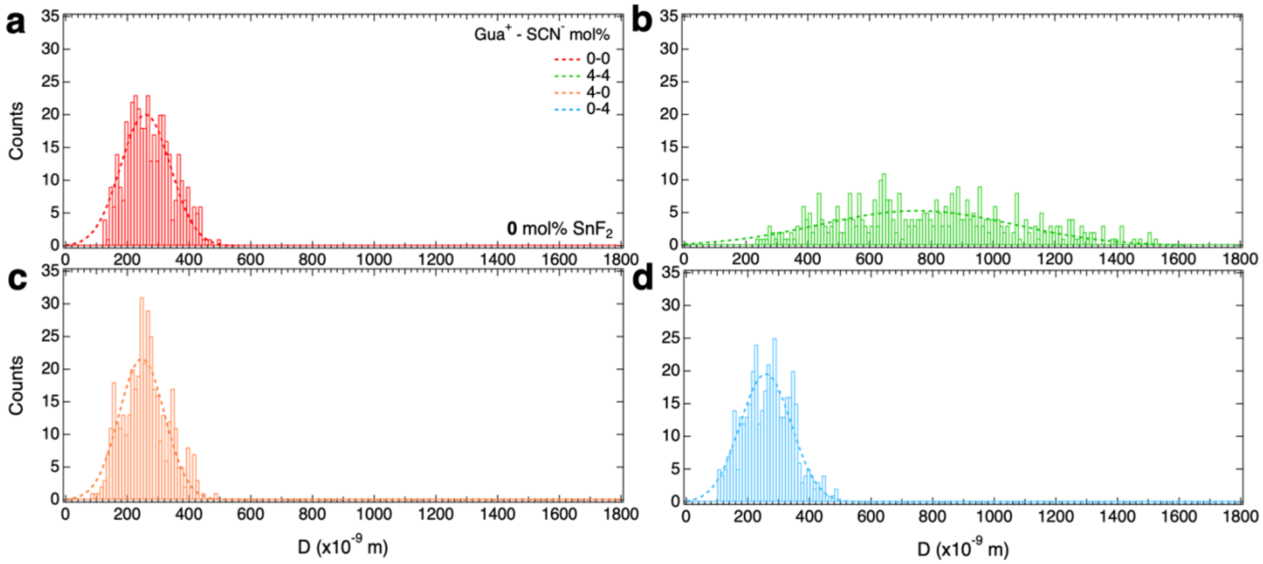

Figure S11: Gaussian distribution of grain size, *D*, in perovskite thin films with 0 or 4 mol% Gua^+ and SCN^- ions and 0 mol% SnF_2.

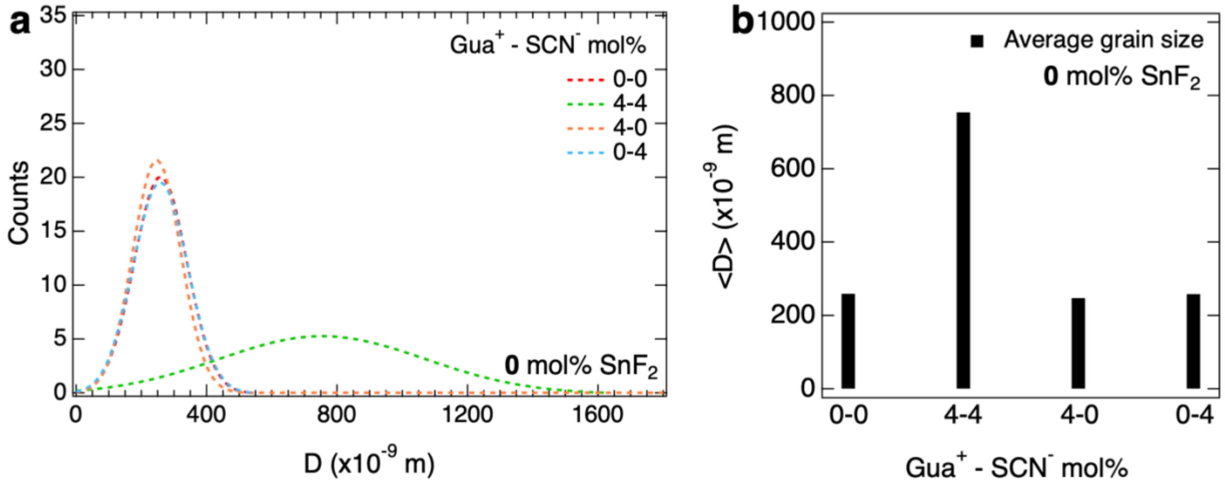

Figure S12: Gaussian distribution of grain size, *D*, and average grain size, <*D*>, in perovskite thin films with 0 or 4 mol% Gua^+ and SCN^- ions and 0 mol% SnF_2.

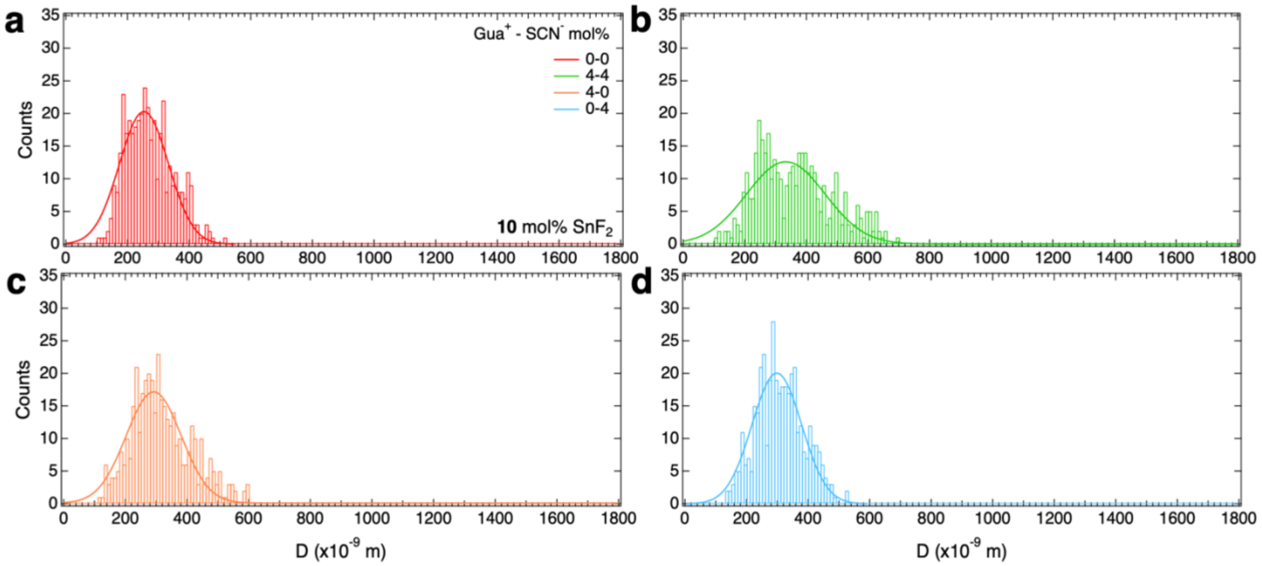

Figure S13: Gaussian distribution of grain size, *D*, in perovskite thin films with 0 or 4 mol% Gua^+ and SCN^- ions and 10 mol% SnF_2.

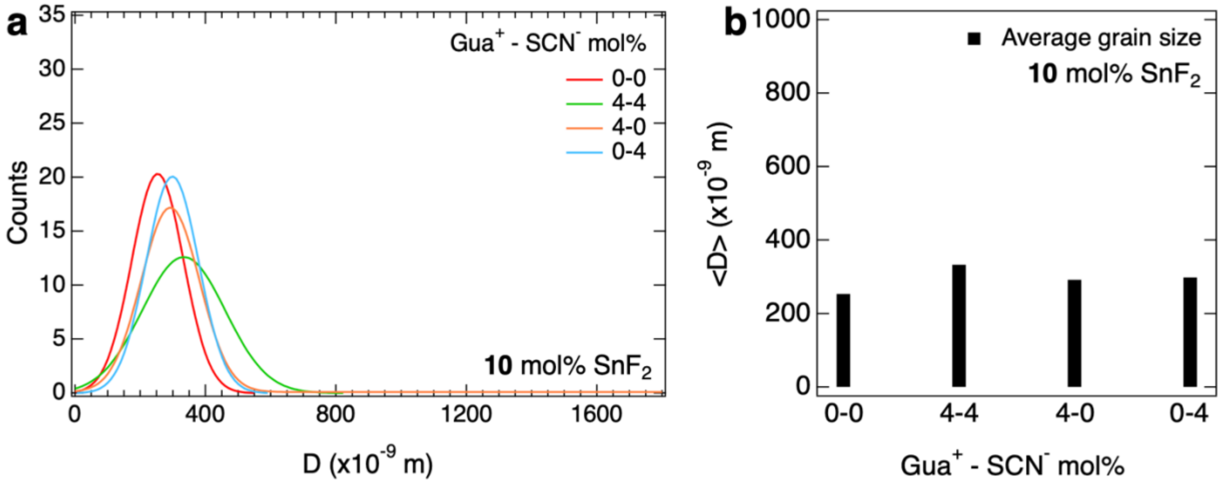

Figure S14: Gaussian distribution of grain size, *D*, and average grain size, <*D*>, in perovskite thin films with 0 or 4 mol% Gua^+ and SCN^- ions and 10 mol% SnF_2.

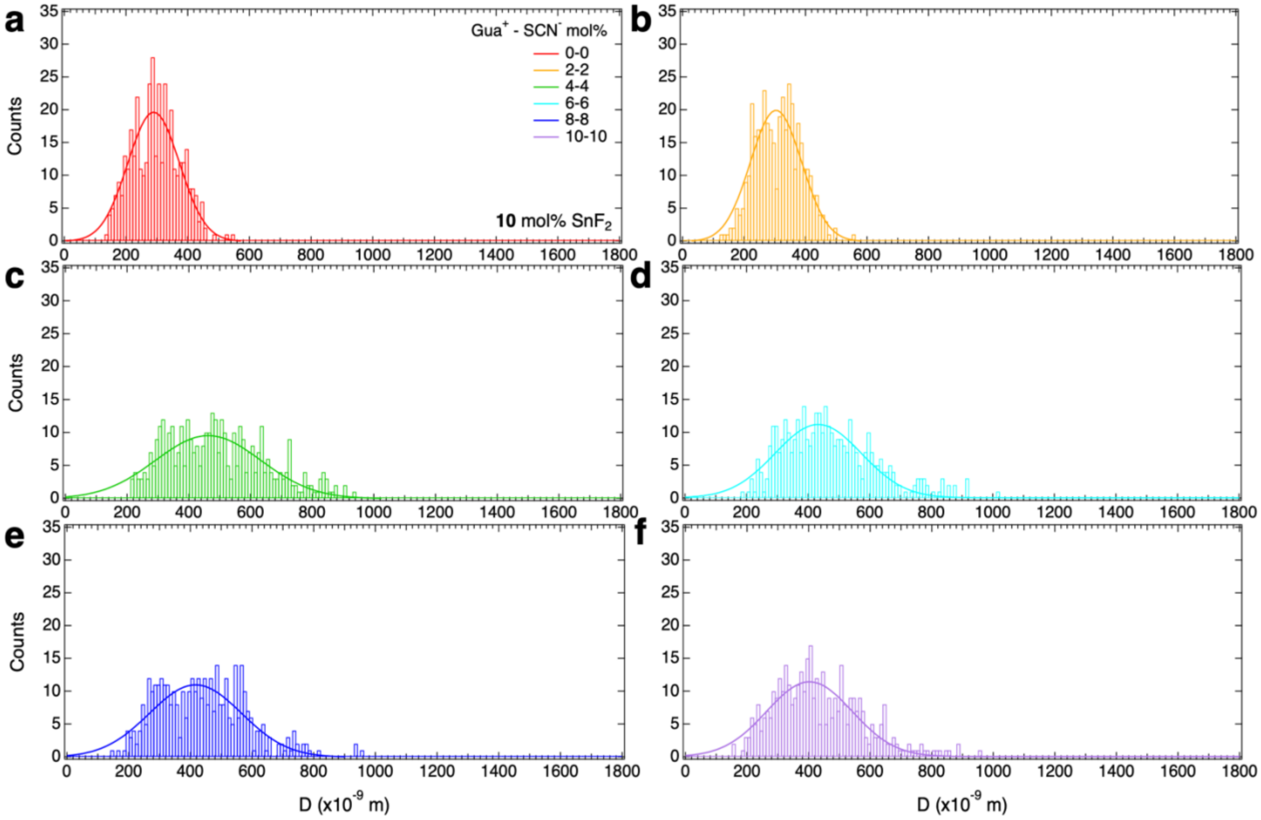

Figure S15: Gaussian distribution of grain size, *D*, in perovskite thin films as a function of varying GuaSCN mol% with 10 mol% SnF_2 addition.

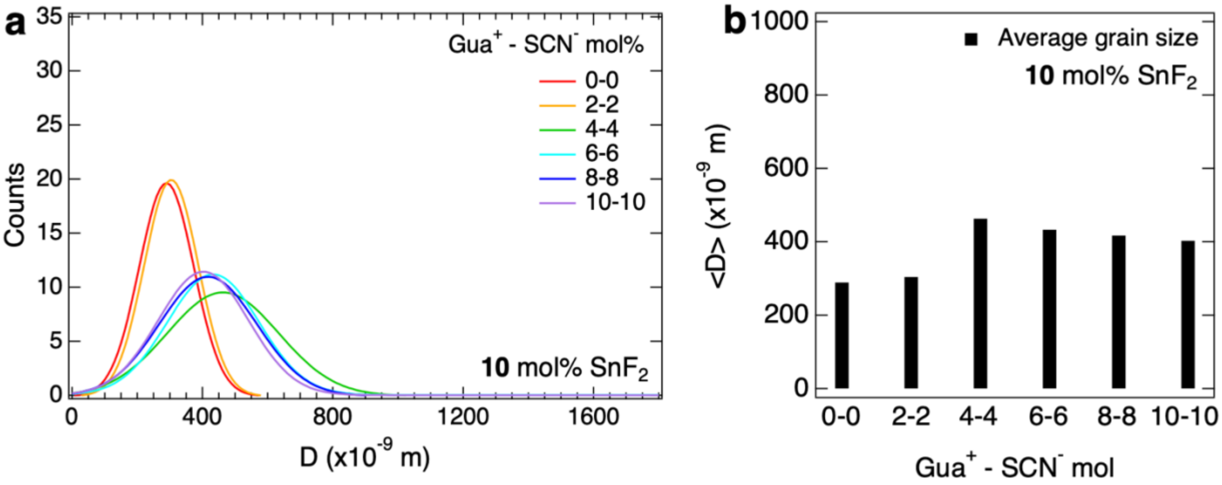

Figure S16: Gaussian distribution of grain size, *D*, and average grain size, <*D*>, in perovskite thin films with varying GuaSCN mol% and 10 mol% SnF_2 addition.

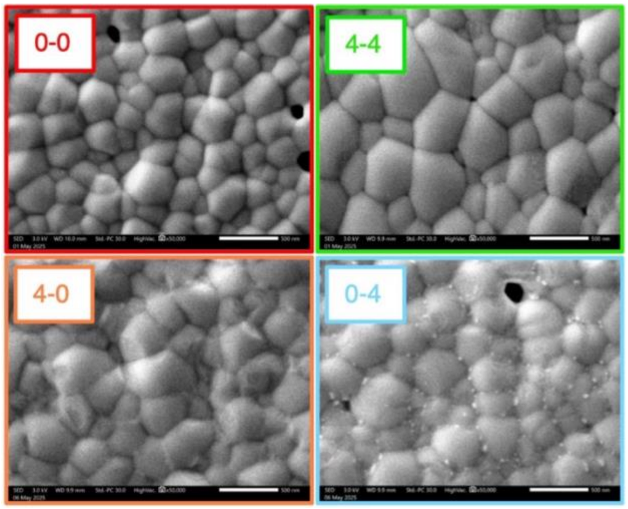

Figure S17: Separate and combined contributions of Gua^{+} and SCN^{-} ions on the microstructural properties by addition of 0 and 4 mol% Gua^{+} and SCN^{-} ions and 10 mol% SnF_2. The SEM images were taken at 50000x magnification.

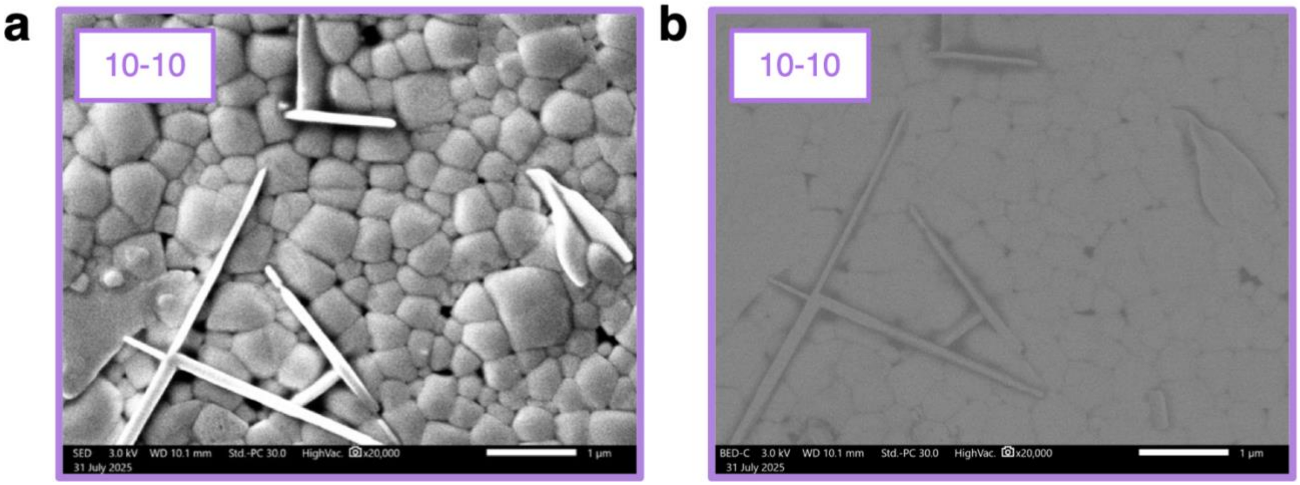

Figure S18: (a) Secondary electrons and (b) back-scattered electrons SEM images of a perovskite film with 10 mol% GuaSCN and 10 mol% SnF_2. Both SEM images were taken at 20000x magnification.

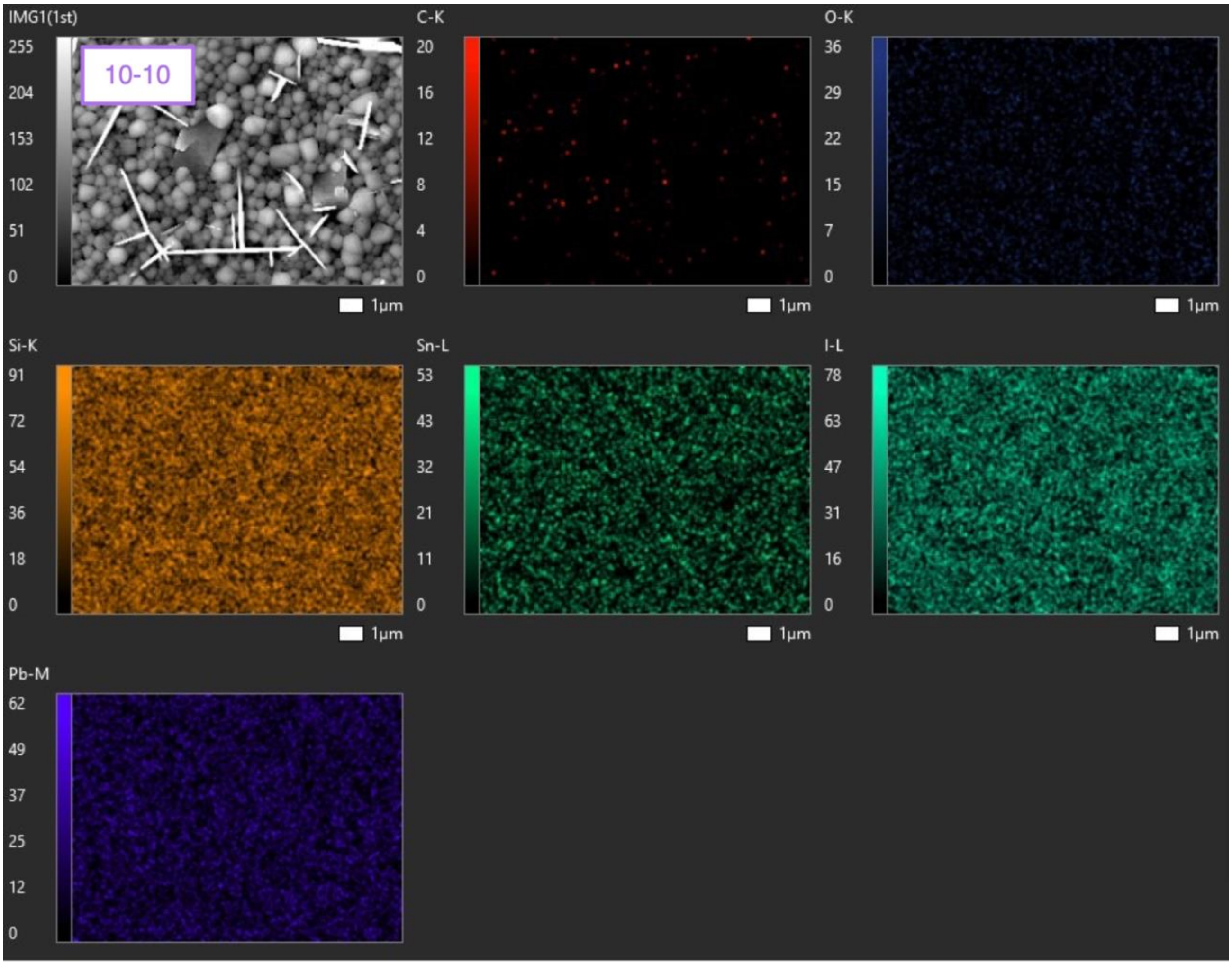

Figure S19: Secondary electrons SEM image and corresponding spatially resolved EDX analysis of C, O, Si, Sn, I and Pb elements performed by SEM of a perovskite film with 10 mol% GuaSCN and 10 mol% SnF_2. For these elemental maps, the analyzed area of the film is ~ 123 μm^2.

X-Ray Diffraction (XRD) – Crystal phase identification and reference XRD pattern comparison

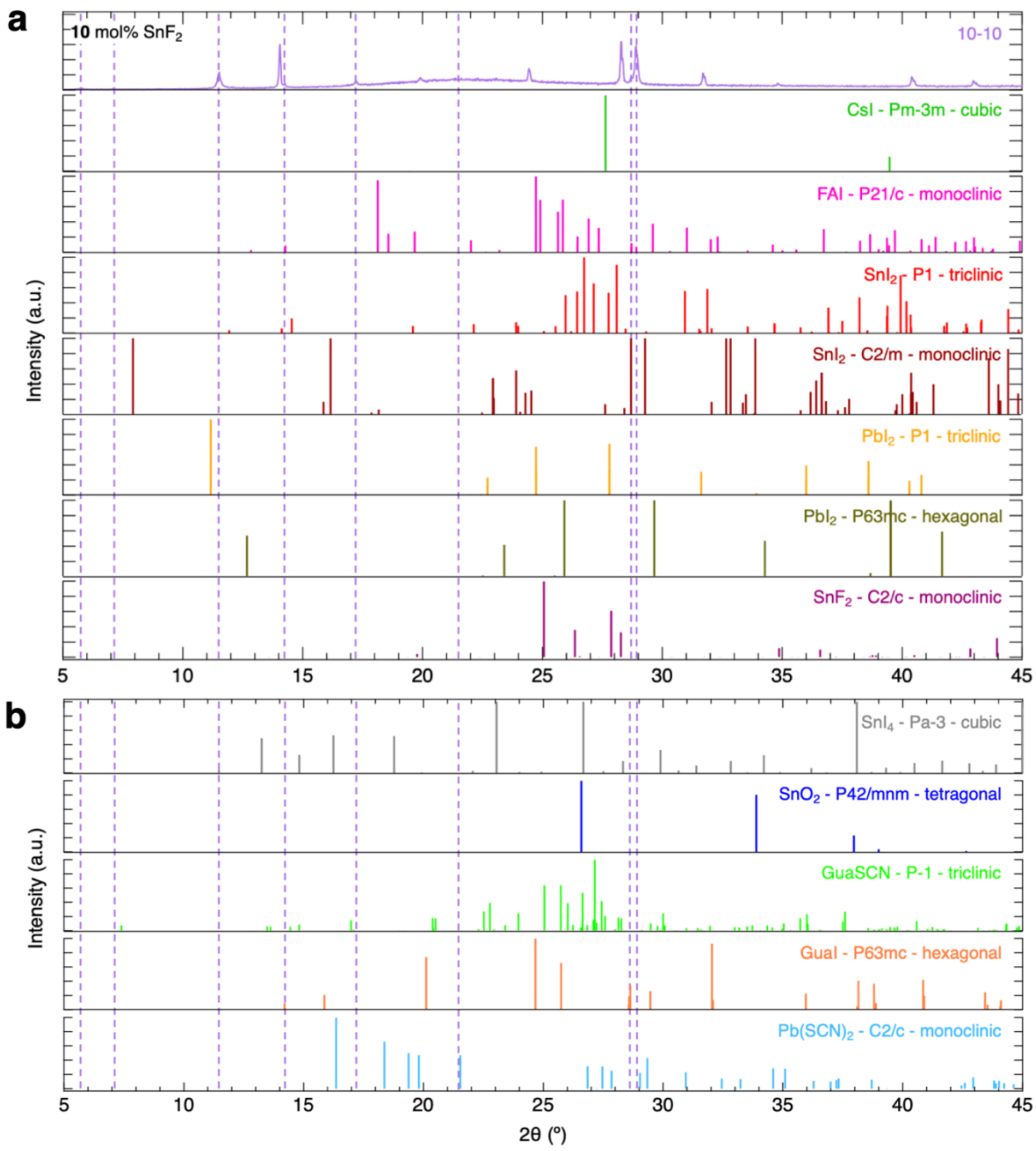

Figure S20: XRD pattern of a perovskite thin film with 10 mol% GuaSCN and 10 mol% SnF_2 addition compared to reference XRD patterns of impurities, such as (a) CsI, FAI, SnI_2, PbI_2, and SnF_2 and (b) SnI_4, SnO_2, GuaSCN, GuaI and $Pb(SCN)_2$. The XRD peaks belonging to the additional phases found in the perovskite films are shown with dark purple dashed lines. The simulated reference patterns of the expected impurities are reported from XPS databases.[1-3]

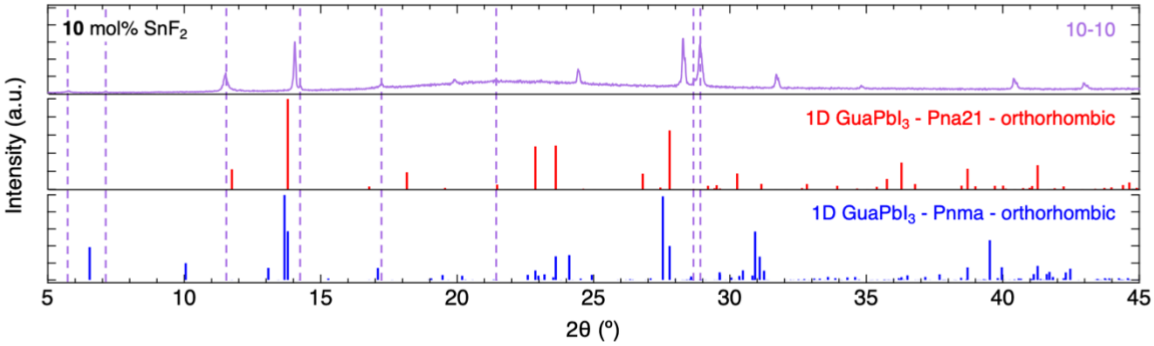

Figure S21: XRD pattern of a perovskite thin film with 10 mol% GuaSCN and 10 mol% SnF_2 addition compared to reference XRD patterns of Gua-containing 1D perovskite phases reported in the literature.[4,5]

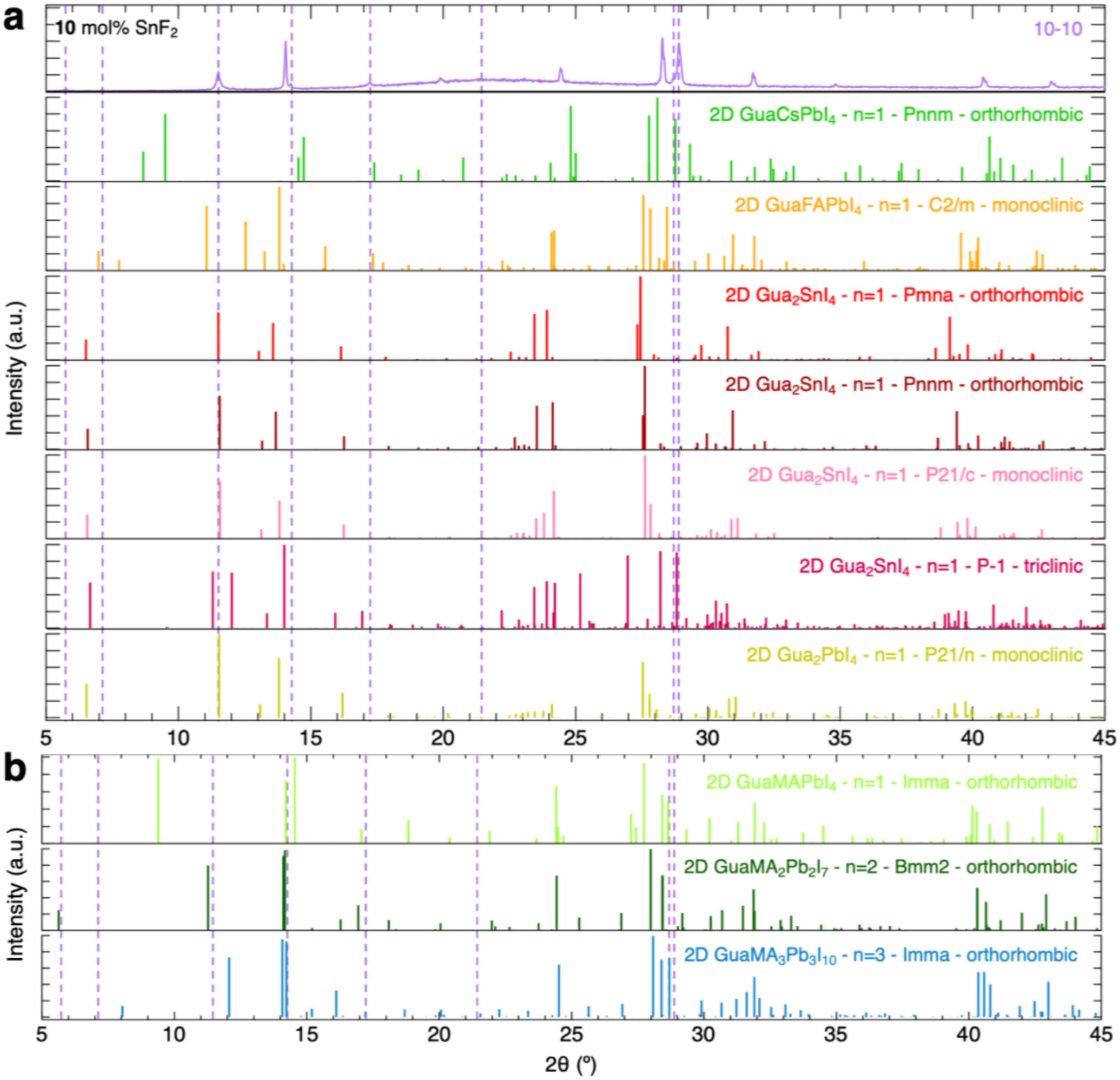

Figure S22: XRD pattern of a perovskite thin film with 10 mol% GuaSCN and 10 mol% SnF_2 addition compared to reference XRD patterns of various Gua-containing 2D perovskite phases reported in the literature.[5-10] The number of inorganic octahedra layers typical for low-dimensional perovskites, *n*, is also shown. In (a), reference XRD patterns of Sn-based and Pb-based 2D phases with *n* = 1 containing only Gua^+ or a mixture of Gua^+, Cs^+ or FA^+ are shown.[5-9] In (b), reference XRD patterns of Pb-based 2D phases with *n* = 1, 2 or 3 containing a mixture of Gua^+ and MA^+ are shown.[10] In detail, *n* is the number of inorganic octahedra layers for low-dimensional perovskites.

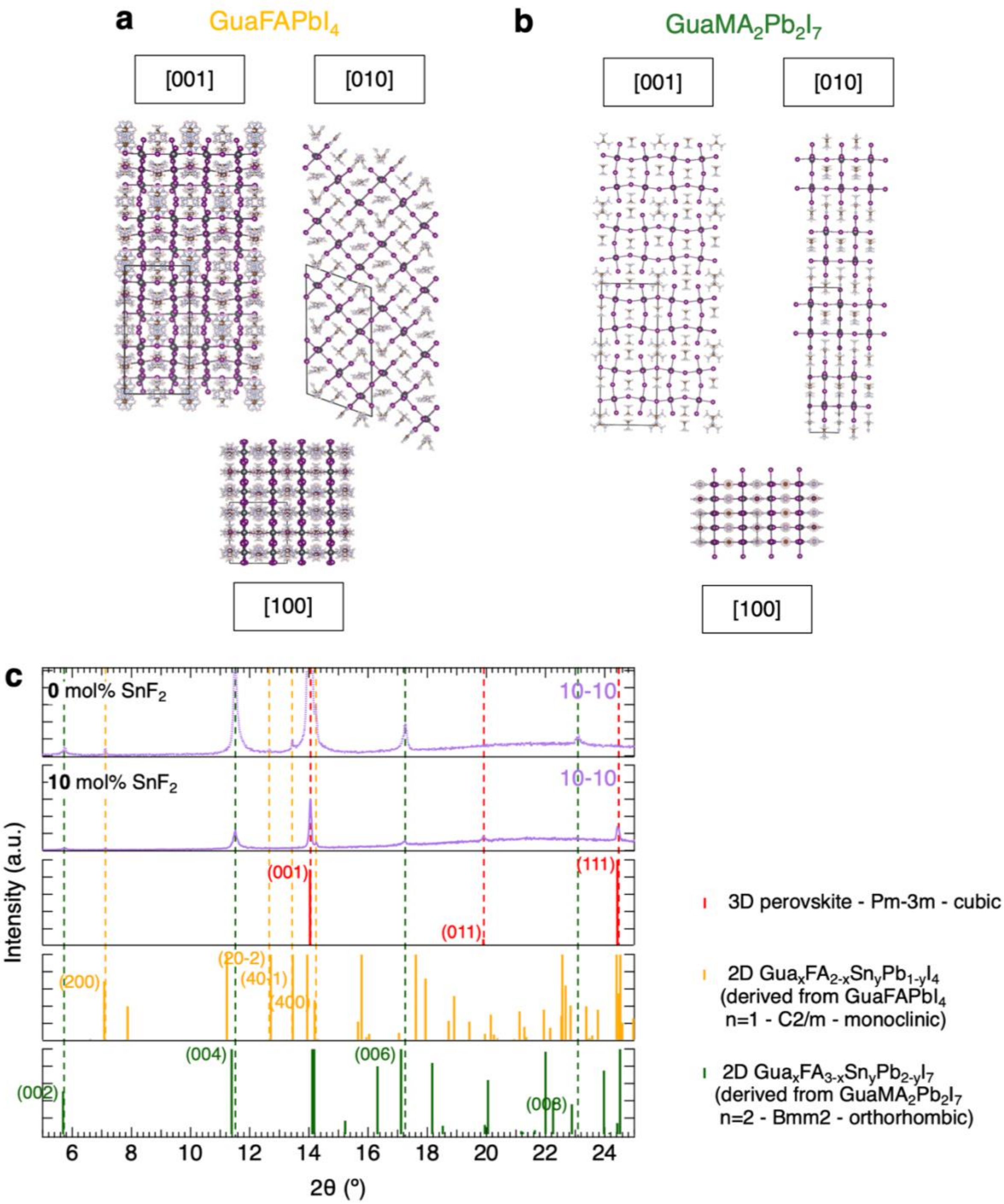

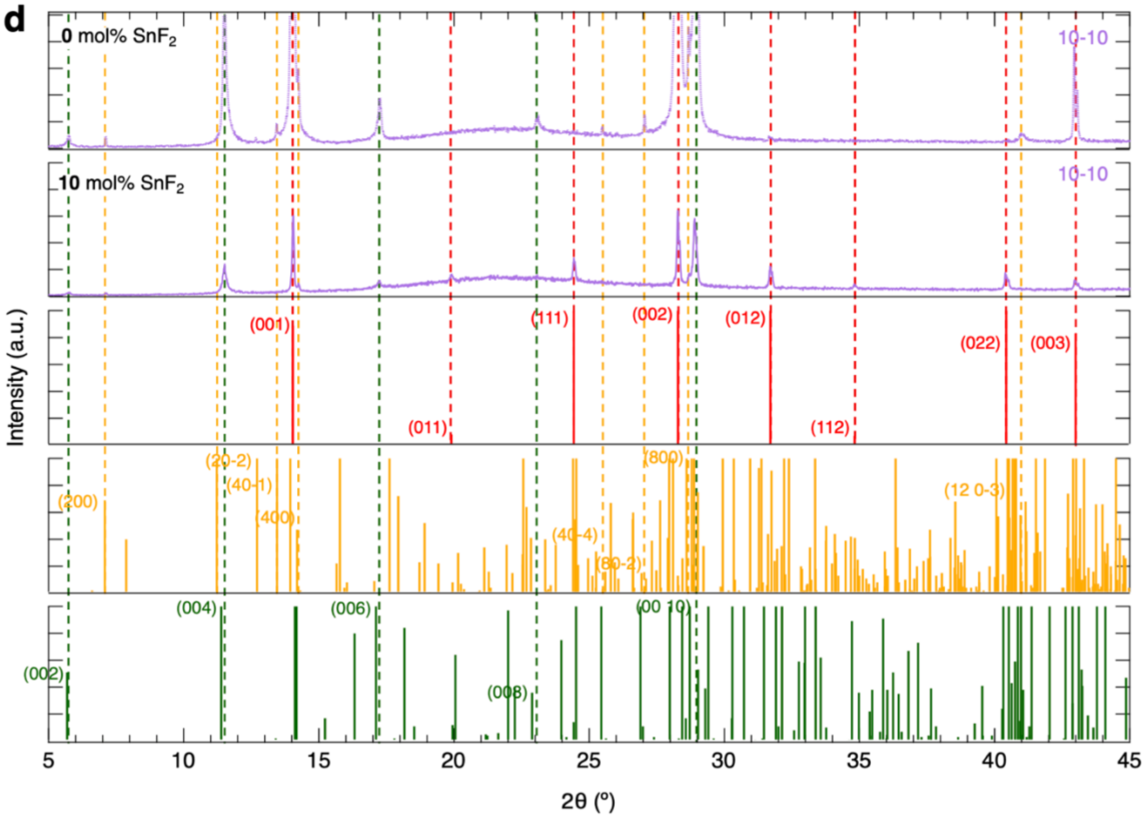

Figure S23: Crystal structures of 2D phases (a) $GuaFAPbI_4$ ("stairs-like" layers n = 1, C2/m, monoclinic) and (b) $GuaMA_2Pb_2I_7$ (n = 2, Bmm2, orthorhombic), where n is the number of inorganic octahedra layers typical for low-dimensional perovskites. The unit cell is delimited by solid black lines. The represented structures are made of 2x2x2 unit cells. The view direction is also shown. (c)(d) XRD pattern of a perovskite thin film with 10 mol% GuaSCN, and 0 or 10 mol% SnF_2 addition compared to reference XRD patterns of a 3D cubic perovskite phase (red dashed lines and Miller indices) and Gua-containing 2D perovskite phases $Gua_xFA_{2-x}Sn_yPb_{1-y}I_4$ and $Gua_xFA_{3-x}Sn_yPb_{2-y}I_7$ (dark yellow and dark green dashed lines and Miller indices, respectively). The unit cells of the simulated reference patterns of $GuaFAPbI_4$ and $GuaMA_2Pb_2I_7$, reported from the literature and shown in **Figure S22a and S22b**,[9,10] were tuned to match the lattice parameters of the phases found in the perovskite films, which we identified as mixtures such as $Gua_xFA_{2-x}Sn_yPb_{1-y}I_4$ and $Gua_xFA_{3-x}Sn_yPb_{2-y}I_7$. In (c), low diffraction angles in the range 2θ = 5-25° are shown, while in (d) a longer range of diffraction angles 2θ = 5-45° are shown.

Table S1: XRD peak position, *i.e.* diffraction angle 2θ, corresponding interplanar distance d_{hkl}, and identified periodicity of d_{hkl}, for the layer with 10 mol% GuaSCN and 0 mol% SnF_2. The periodic repetition of d_{hkl} and relative crystal planes attributed to $Gua_xFA_{2-x}Sn_yPb_{1-y}I_4$ and $Gua_xFA_{3-x}Sn_yPb_{2-y}I_7$ (derived from tuning $GuaFAPbI_4$ and $GuaMA_2Pb_2I_7$ references, respectively) are shown (in dark yellow and dark green, respectively). We identified for $Gua_xFA_{2-x}Sn_yPb_{1-y}I_4$ three main orientations, [200], [20-2] and [40-1], and for $Gua_xFA_{3-x}Sn_yPb_{2-y}I_7$ one preferential orientation, [002] (direction of stacking).

2θ (º)	***d_{hkl} (nm)***	***Periodicity of d_{hkl}***
5.74	1.538	w (002)
7.11	1.242	x (200)
11.51	0.768	w/2 (004)
12.67	0.698	y (20-2)
13.44	0.658	z (40-1)
14.24	0.621	x/2 (400)
17.25	0.514	w/3 (006)
23.09	0.385	w/4 (008)
25.48	0.349	y/2 (40-4)
27.05	0.329	z/2 (80-2)
28.68	0.311	x/4 (800)
28.94	0.308	w/5 (00 10)
41.02	0.220	z/3 (12 0-3)

Table S2: XRD peak position, *i.e.* diffraction angle 2θ, corresponding interplanar distance d_{hkl}, and identified periodicity of d_{hkl}, for the layer with 10 mol% GuaSCN and 10 mol% SnF_2. The periodic repetition of d_{hkl} and relative crystal planes attributed to $Gua_xFA_{2-x}Sn_yPb_{1-y}I_4$ and $Gua_xFA_{3-x}Sn_yPb_{2-y}I_7$ (derived from tuning $GuaFAPbI_4$ and $GuaMA_2Pb_2I_7$ references, respectively) are shown (in dark yellow and dark green, respectively). We identified for $Gua_xFA_{2-x}Sn_yPb_{1-y}I_4$ one main orientation, [200], and for $Gua_xFA_{3-x}Sn_yPb_{2-y}I_7$ one preferential orientation, [002] (direction of stacking).

2θ (º)	***d_{hkl} (nm)***	***Periodicity of d_{hkl}***
5.74	1.538	w (002)
7.12	1.241	x (200)
11.51	0.768	w/2 (004)
14.25	0.621	x/2 (400)
17.22	0.514	w/3 (006)
23.05	0.385	w/4 (008)
28.70	0.311	x/4 (800)
28.87	0.309	w/5 (00 10)

X-ray Photoelectron Spectroscopy (XPS) – Elemental composition and depth profiling

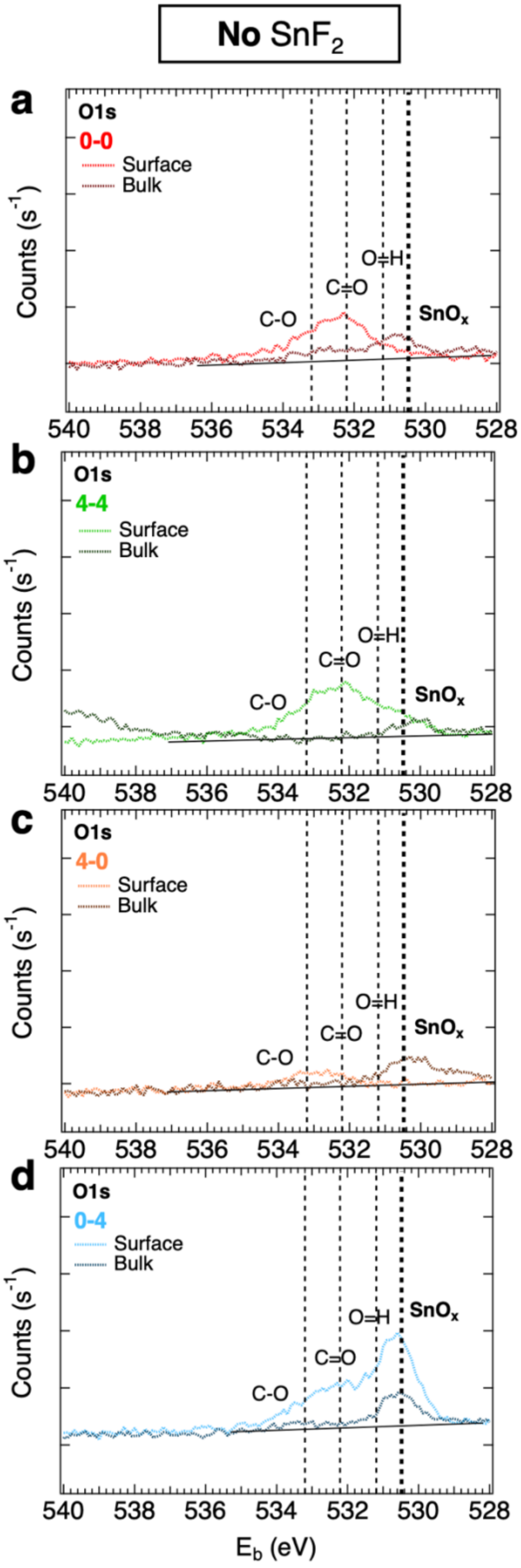

Figure S24: Surface and bulk XPS scans of O1s orbitals of perovskite thin film with (a) 0 mol% Gua^+ and/or SCN^-, (b) 4 mol% GuaSCN, (c) 4 mol% Gua^+ or (d) 4 mol% SCN^- + 0 mol% SnF_2 addition. The reference for C-O, C=O, O-H and SnO_x are just indicative and were reported from the literature.[11,1]

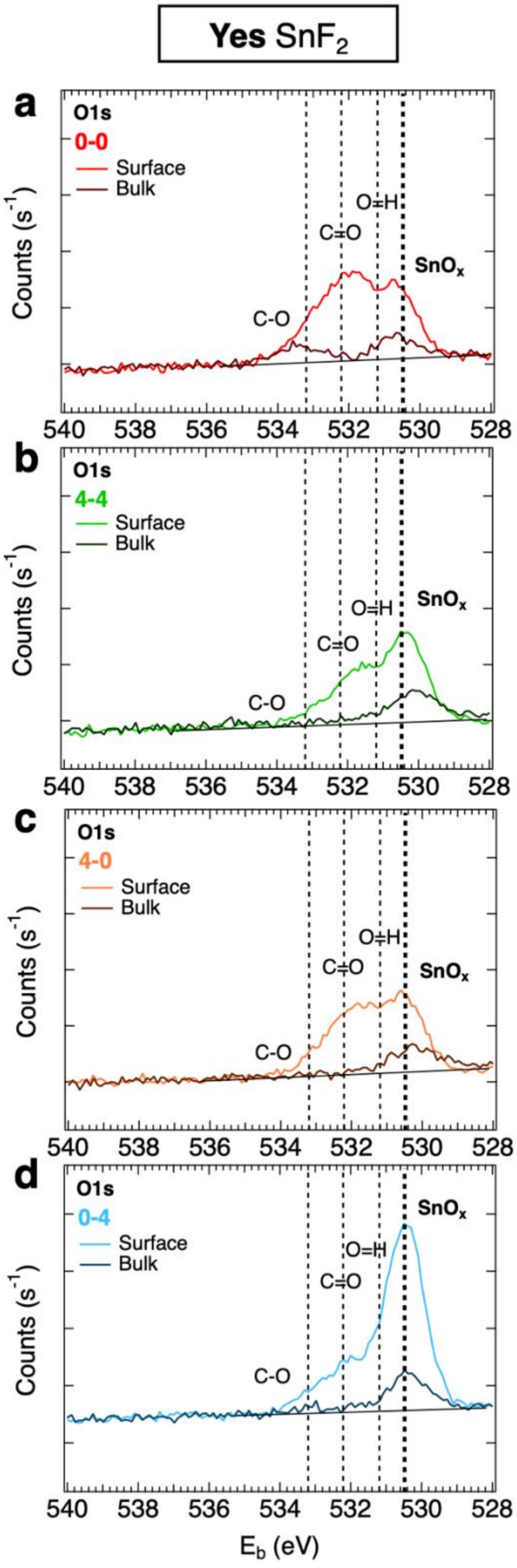

Figure S25: Surface and bulk XPS scans of O1s orbitals of perovskite thin film with (a) 0 mol% Gua^+ and/or SCN^-, (b) 4 mol% GuaSCN, (c) 4 mol% Gua^+ or (d) 4 mol% SCN^- + 10 mol% SnF_2 addition. The reference for C-O, C=O, O-H and SnO_x were reported from the literature.[11,12]

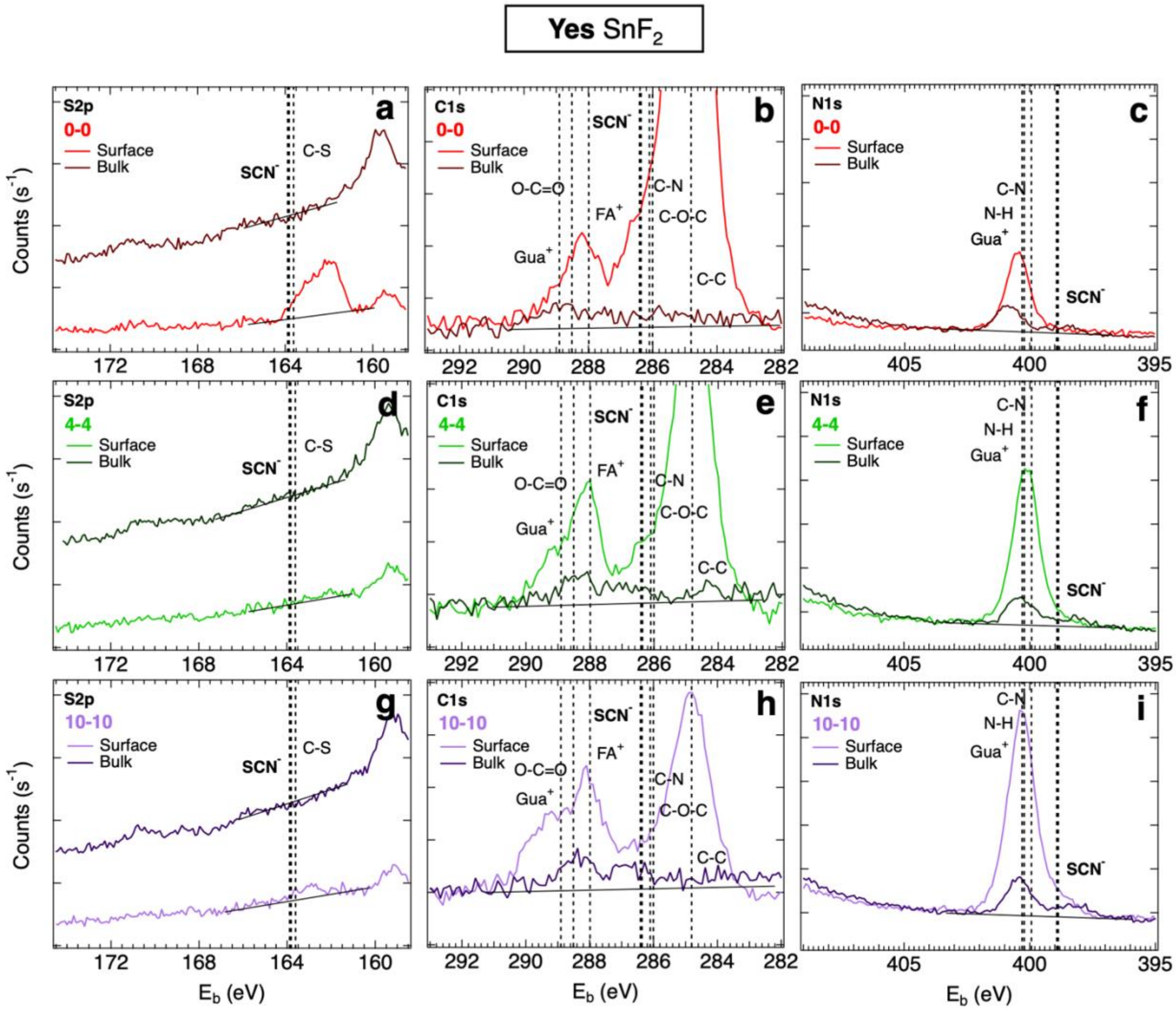

Figure S26: Surface and bulk XPS scans of S2p, C1s and N1s orbitals in perovskite thin film with 0, 4 or 10 mol% GuaSCN + 10 mol% SnF_2 addition. The reference for SCN^- and C-S in the S2p scans were reported from the literature,[14] as well for the references for Gua^+, FA^+, SCN^- and C-C in the C1s scans.[11,13,14] The reference for N-H, C-N and SCN^- in the N1s scans were also reported from publications.[14,15]

XRD, SEM and TRMC - Structural and Charge Carrier Transport properties of perovskite layers with addition of 0 or 10 mol% GuaSCN and 0 mol% SnF_2

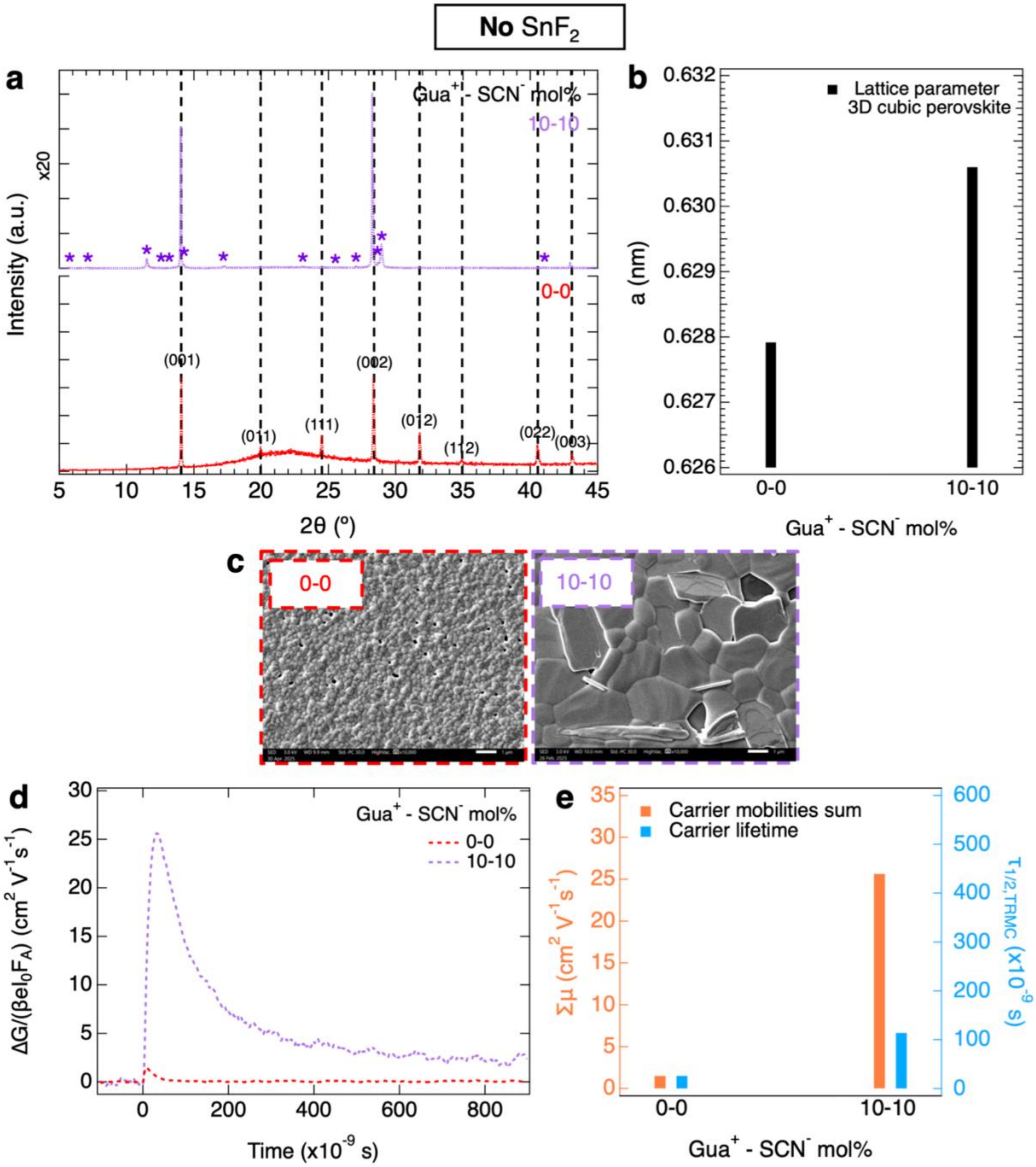

Figure S27: Structural and charge carrier transport properties of perovskite thin film by addition of 0 or 10 GuaSCN and 0 mol% SnF_2. Note the multiplication factor x 20 on the XRD intensity axis for the layer with 10 mol% GuaSCN and 0 mol% SnF_2 additions. The XRD shows peaks corresponding to a 3D cubic perovskite phase (black dashed lines and Miller indices) and to additional Gua-based 2D perovskite phases

for the addition of ≥ 6 mol% GuaSCN ((*) markers). The lattice parameter, *a*, of the 3D cubic perovskite phase is also shown. The SEM images were taken at 10000x magnification.

Pseudo J-V curves parameters

Table S3: Parameters of the pseudo J-V curves derived from the microwave-based quasi-Fermi level splitting (QFLS) measurements of the reference film from deposition B with 10 mol% SnF_2 w.r.t. SnI_2 addition. The parameters shown are the pseudo current density, *pJ,* pseudo voltage, *pV*, pseudo power, *pP*, and pseudo fill factor at the pseudo maximum power point, *pFF*.

**Deposition B**: With SnF_2; With GuaSCN or GuaI or $Pb(SCN)_2$				
Gua^+ - SCN^- mol% : 0-0	pJ (mA cm^{-2})	pV (V)	pP (W)	pFF (-)
	-4.11	0.76	-3.14	
	8.51	0.75	6.42	
	24.92	0.74	18.34	
	31.23	0.72	22.33	
	32.87	0.70	22.96	
	33.50	0.70	23.34	0.91
	33.66	0.68	22.84	
	33.72	0.66	22.23	
	33.74	0.64	21.50	
	33.75	0.63	21.15	
	33.75	0	0	

Table S4: Parameters of the pseudo J-V curves derived from the microwave-based quasi-Fermi level splitting (QFLS) measurements of the reference film from deposition B with 4 mol% GuaSCN w.r.t. (CsI+FAI) and 10 mol% SnF_2 w.r.t. SnI_2 addition. The parameters shown are the pseudo current density, *pJ,* pseudo voltage, *pV*, pseudo power, *pP*, and pseudo fill factor at the pseudo maximum power point, *pFF*.

**Deposition B**: With SnF_2; With GuaSCN or GuaI or $Pb(SCN)_2$				
Gua^+ - SCN^- mol% : 4-4	pJ (mA cm^{-2})	pV (V)	pP (W)	pFF (-)
	-4.11	0.81	-3.32	
	8.51	0.80	6.82	
	24.92	0.78	19.44	
	31.23	0.76	23.60	
	32.87	0.73	24.03	0.88
	33.50	0.71	23.75	
	33.66	0.69	23.09	
	33.72	0.66	22.24	
	33.74	0.64	21.46	
	33.75	0.62	20.94	
	33.75	0	0	

Table S5: Parameters of the pseudo J-V curves derived from the microwave-based quasi-Fermi level splitting (QFLS) measurements of the reference film from deposition B with 4 mol% GuaI w.r.t. (CsI+FAI) and 10 mol% SnF_2 w.r.t. SnI_2 addition. The parameters shown are the pseudo current density, *pJ,* pseudo voltage, *pV*, pseudo power, *pP*, and pseudo fill factor at the pseudo maximum power point, *pFF*.

**Deposition B**: With SnF_2; With GuaSCN or GuaI or $Pb(SCN)_2$				
Gua^+ - SCN^- mol% : 4-0	pJ (mA cm^{-2})	pV (V)	pP (W)	pFF (-)
	-4.11	0.77	-3.18	
	8.51	0.76	6.49	
	24.92	0.74	18.41	
	31.23	0.71	22.31	
	32.87	0.70	22.85	0.88
	33.50	0.67	22.56	
	33.66	0.65	21.81	
	33.72	0.63	21.21	
	33.75	0	0	

Table S6: Parameters of the pseudo J-V curves derived from the microwave-based quasi-Fermi level splitting (QFLS) measurements of the reference film from deposition B with 2 mol% $Pb(SCN)_2$ w.r.t. (CsI+FAI) and 10 mol% SnF_2 w.r.t. SnI_2 addition. The parameters shown are the pseudo current density, *pJ,* pseudo voltage, *pV*, pseudo power, *pP*, and pseudo fill factor at the pseudo maximum power point, *pFF*.

**Deposition B**: With SnF_2; With GuaSCN or GuaI or $Pb(SCN)_2$				
Gua^+ - SCN^- mol% : 0-4	pJ (mA cm^{-2})	pV (V)	pP (W)	pFF (-)
	-4.11	0.77	-3.15	
	8.51	0.76	6.44	
	24.92	0.74	18.32	
	31.23	0.71	22.23	
	32.87	0.70	22.88	0.88
	33.50	0.68	22.63	
	33.66	0.65	22.01	
	33.72	0.64	21.47	
	33.74	0.62	20.85	
	33.75	0	0	

Data Availability Statement

Data for this article, including the raw data for the XRD, UV-Vis-NIR spectroscopy, SSMC, TRMC and XPS results, as well as the SEM images and EDX analysis data, are available at 4TU.ResearchData at 10.4121/1a71eea7-82a9-4b4f-965a-9df24c4706df.

References

(1) Cambridge Crystallographic Data Centre / Cambridge Structural Database (CSD), 2025, https://www.ccdc.cam.ac.uk/solutions/software/csd/, (accessed April 2025).

(2) Crystallography Open Database / Crystallography Open Database (COD), 2025, https://www.crystallography.net/cod/index.php, (accessed April 2025).

(3) Materials Project / Materials Project, 2020, https://legacy.materialsproject.org, (accessed April 2025).

(4) A. D. Jodlowski, A. Yépez, R. Luque, L. Camacho and G. De Miguel, *Angew. Chem. Int. Ed.*, 2016, **55**, 14972–14977. DOI: 10.1002/anie.201607397

(5) P. Wu, D. Li, S. Wang and F. Zhang, *Mater. Chem. Front.*, 2023, **7**, 2507–2527. DOI: 10.1039/d2qm01315k

(6) O. Nazarenko, M. R. Kotyrba, M. Wörle, E. Cuervo-Reyes, S. Yakunin and M. V. Kovalenko, *Inorg. Chem.*, 2017, **56**, 11552–11564. DOI: 10.1021/acs.inorgchem.7b01204

(7) M. Daub, C. Haber and H. Hillebrecht, *Eur. J. Inorg. Chem.*, 2017, **2017**, 1120–1126. DOI: 10.1002/ejic.201601499

(8) M. Szafrański and A. Katrusiak, *Phys. Rev. B,* 2000, **61**, 1026–1035. DOI: 10.1103/physrevb.61.1026

(9) O. Nazarenko, M. R. Kotyrba, S. Yakunin, M. Aebli, G. Rainò, B. M. Benin, M. Wörle and M. V. Kovalenko, *J. Am. Chem. Soc.*, 2018, **140**, 3850–3853. DOI: 10.1021/jacs.8b00194

(10) C. M. M. Soe, C. C. Stoumpos, M. Kepenekian, B. Traoré, H. Tsai, W. Nie, B. Wang, C. Katan, R. Seshadri, A. D. Mohite, J. Even, T. J. Marks and M. G. Kanatzidis, *J. Am. Chem. Soc.*, 2017, **139**, 16297–16309. DOI: 10.1021/jacs.7b09096

(11) *Table of Elements | Thermo Fisher Scientific - US*. (n.d.). https://www.thermofisher.com/nl/en/home/materials-science/learning-center/periodic-table.html

(12) A. Treglia, M. Prato, C. J. Wu, E. L. Wong, I. Poli and A. Petrozza, *Adv. Funct. Mater.*, 2024, **34**, 2406954. DOI: 10.1002/adfm.202406954

(13) J. Tong, Z. Song, D. H. Kim, X. Chen, C. Chen, A. F. Palmstrom, P. F. Ndione, M. O. Reese, S.P. Dunfield, O. G. Reid, J. Liu, F. Zhang, S. P. Harvey, Z. Li, S. T. Christensen, G. Teeter, D. Zhao, M. M. Al-Jassim, M. F. A. M. van Hest, M. C. Beard, S.E. Shaheen, J. J. Berry, Y. Yan and K. Zhu, *Science*, 2019, **364**, 475–479. DOI: 10.1126/science.aav7911

(14) B. Wang, S. Nam, S. Limbu, J. Kim, M. Riede and D. D. C. Bradley, *Adv. Electron. Mater.*, 2022, **8**, 2101253. DOI: 10.1002/aelm.202101253

(15) A.D. Jodlowski, C. Roldán-Carmona, G. Grancini, M. Salado, M. Ralaiarisoa, S. Ahmad, N. Koch, L. Camacho, G. De Miguel and M. K. Nazeeruddin, *Nat. Energy*, 2017**, 2**, 972–979. DOI: 10.1038/s41560-017-0054-3
